# Circadian Photoentrainment in Mice and Humans

**DOI:** 10.3390/biology9070180

**Published:** 2020-07-21

**Authors:** Russell G. Foster, Steven Hughes, Stuart N. Peirson

**Affiliations:** Sleep & Circadian Neuroscience Institute (SCNi), Nuffield Department of Clinical Neurosciences, Sir William Dunn School of Pathology, Oxford Molecular Pathology Institute, South Parks Road, University of Oxford, Oxford OX1 3RF, UK; steven.hughes@ndcn.ox.ac.uk (S.H.); stuart.peirson@eye.ox.ac.uk (S.N.P.)

**Keywords:** circadian, entrainment, human, melanopsin (OPN4), mouse, photoreceptor

## Abstract

Light around twilight provides the primary entrainment signal for circadian rhythms. Here we review the mechanisms and responses of the mouse and human circadian systems to light. Both utilize a network of photosensitive retinal ganglion cells (pRGCs) expressing the photopigment melanopsin (OPN4). In both species action spectra and functional expression of OPN4 in vitro show that melanopsin has a λ_max_ close to 480 nm. Anatomical findings demonstrate that there are multiple pRGC sub-types, with some evidence in mice, but little in humans, regarding their roles in regulating physiology and behavior. Studies in mice, non-human primates and humans, show that rods and cones project to and can modulate the light responses of pRGCs. Such an integration of signals enables the rods to detect dim light, the cones to detect higher light intensities and the integration of intermittent light exposure, whilst melanopsin measures bright light over extended periods of time. Although photoreceptor mechanisms are similar, sensitivity thresholds differ markedly between mice and humans. Mice can entrain to light at approximately 1 lux for a few minutes, whilst humans require light at high irradiance (>100’s lux) and of a long duration (>30 min). The basis for this difference remains unclear. As our retinal light exposure is highly dynamic, and because photoreceptor interactions are complex and difficult to model, attempts to develop evidence-based lighting to enhance human circadian entrainment are very challenging. A way forward will be to define human circadian responses to artificial and natural light in the “real world” where light intensity, duration, spectral quality, time of day, light history and age can each be assessed.

## 1. Shedding Light on the Clock—The Phase Response Curve

To be of any value, an endogenous circadian clock must be set to local time. The majority of circadian clocks utilize a solar-based mechanism as the primary means to synchronize (entrain) the biological day to the astronomical day. For more than four billion years, the changes in the quality and quantity of light at twilight have been the main time-giver or “zeitgeber” that enables entrainment for life on Earth [1]. Circadian clocks are not exactly 24 h (hence the term: circa/about and dies/day), and in this regard resemble an old mechanical grandfather clock which needs a slight daily adjustment to make sure the clock is set to the “real” astronomical day. Without this daily re-setting, the internal day would soon drift and be out of alignment with the environmental day/night cycle. In multicellular organisms, a master clock is usually entrained to the external light/dark cycle, and then acts in-turn to entrain multiple circadian oscillators throughout the rest of the body (peripheral clocks). Although light is the primary zeitgeber for the circadian system of most organisms, it is not the only zeitgeber. Most, if not all cells within multicellular organisms possess the ability to express a circadian rhythm, and these independent clocks can be regulated by a variety of different signals. These peripheral clocks then drive countless behavioral, physiological and biochemical outputs [2]. Thus, there is a complex circadian network within an individual that is regulated by a hierarchy of zeitgebers which “fine-tune” performance to the varied demands of the solar cycle.

**Figure 1:**
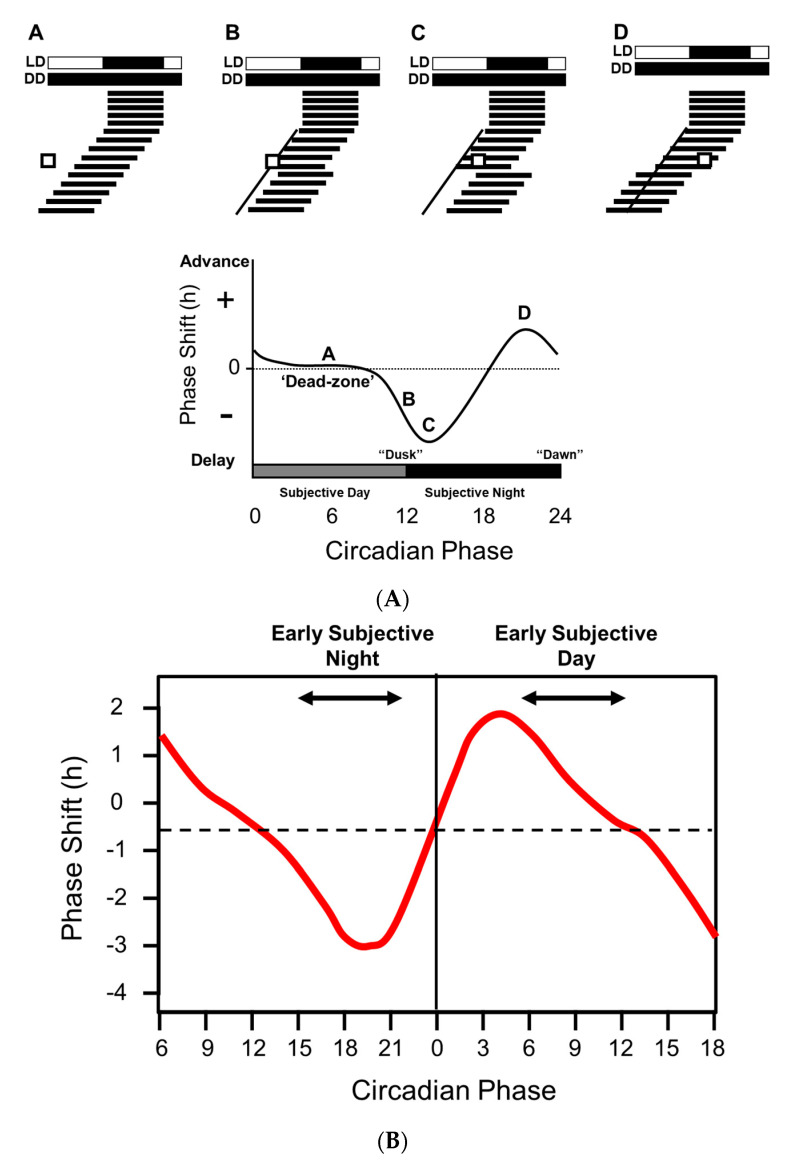
(**A**): phase response curve (PRC) for a nocturnal animal such as a mouse. In the upper part of this figure (A–D) the light/dark cycle is shown and the dark line illustrates the duration of activity (also called “alpha”) on subsequent days. For the first four days the animal is kept under a light/dark cycle of 12 h of light and 12 h of dark (L:D 12:12). On day 5, the lights were switched off and the animal was kept under constant darkness (DD), and it freeran with a period slightly shorter than 24 h. To provide reference points under freerunning conditions, activity onset in a nocturnal animal is termed “circadian time 12” or CT 12. The CT 0–12 is considered as the “subjective day,” and CT 12–24 is considered “subjective night.” If the animal is exposed to a single one-hour pulse of light during its subjective circadian day, as shown in (A), there is usually no or little phase shifting effect on the freerunning rhythm. This is called the “dead zone.” At (B) the light pulse is given early in the subjective night, the effect is to start activity slightly later the next day (a delaying phase shift). In (C) the light exposure is later into the night and there is an increased delaying effect the following day. When light is given during the second half of the night (D), the effect is to advance the freerunning rhythm. If the phase shifts (A–D) are plotted against the circadian time the result produces a phase response curve (PRC). (**B**): One version of the human phase response curve (PRC) derived from human subjects [4]. In this figure, phase advances (positive values) and delays (negative values) have been plotted against the timing of light exposure relative to the measured phase of melatonin, which, in humans, is frequently used as a routine measure of circadian phase. The light “pulse” consisted of 6.7 h bright light exposure alternating between 6 min fixed gaze (approximately 10,000 lux) and free gaze (approximately 5000–9000 lux) exposures. Redrawn from Khalsa et.al. 2003. See text for details.

If animals are maintained under constant darkness and then exposed to a discrete pulse of light at varied times over the subjective day, the shifting (phase shifting) effects on the endogenous clock (freerunning rhythm) vary [3].) Note: Circadian Time (CT) is a standardized notation of the 24 h phase of a circadian cycle representing an estimation of the organism’s subjective time. Light delivered during subjective daytime has a minimal effect. By contrast, light delivered during the first six hours of the subjective night (CT 12–18) cause a phase delay—the animal will start its activity later the following day, whilst light exposure during the second half of the subjective night and towards morning (CT 18–24) will advance activity onset. These differential effects of light are described by the “phase response curve” or PRC. Figure 1A illustrates how a phase response curve (PRC) is generated for a nocturnal animal such as a mouse.

Remarkably, the PRCs of all organisms appear broadly similar, with light exposure between CT 12 and 18 causing a delay in activity onset the next day, and light delivered between CT 18 and 24 generating an advance. The exact shape the PRC is species specific; some have small delays and big advances (typical of diurnal species) whilst others have large delays and small advances (typical of nocturnal species) [5].

There is some controversy regarding the human PRC. Some researchers suggest that humans, like most other animals, have a “dead zone” and that there are no significant phase-shifting effects of light during the day; e.g., [6] (Figure 1A). In contrast, other researchers are strongly of the view that light exposure during the day will contribute to circadian entrainment [7] (Figure 1B). A key issue may be the methods used to define the human PRC, shown in Figure 1B, which were markedly different from those used in rodents. For example, Khalsa and colleagues [4], maintained subjects under a constant routine (CR, also see [8]) of dim light (approximately 2–7 lux) consisting of sustained imposed wakefulness, with the subject maintained in a partly reclining posture for the entire period. Snacks and fluids were provided hourly to maintain an evenly distributed calorie and liquid intake. The phase shifting stimulus consisted of 6.7 h of bright light exposure consisting of 6 min fixed gaze (approximately 10,000 lux) alternating with free gaze (approximately 5000–9000 lux). Such a duration of light exposure (6.7 h) is in marked contrast to the durations used for animal studies, which are much shorter and in the order of minutes. [3]. It should also be emphasized that CR conditions maintain non-photic zeitgebers, and in particular calorie intake, at a constant level. This is not the case for animal studies, where food and feeding behavior could influence peripheral clocks (see below), and potentially provide feedback to the central circadian pacemaker (see below) and the hypothalamus in a way that may influence the PRC. As a result, a direct comparison between rodent and human PRCs is complex based upon these divergent methodologies.

In addition to the discussion relating to the presence or absence of a “dead zone” (Figure 1A vs. Figure 1B), two types of PRC have been described. Type 1 PRCs have a low amplitude, with phase shifts of no more than a few hours, as illustrated in Figure 1A, whilst type 0 PRCs are high amplitude with phase shifts as large as 12 h [5]. Again, there is some controversy in humans regarding the possession of a type 1 vs. a type 0 PRC [9]. Both have been reported in humans, but in the case of the type 0 response, this was achieved by delivering three consecutive cycles of 5 h of bright light (7000–10,000 lux) [10]. Whether such a multiple-pulse PRC can truly be classified as a type 0 has been questioned by several researchers; e.g., Beersma and Daan [9].

Regardless of the form of the PRC, overall one can conclude that light at dusk and dawn acts to push and pull the freerunning rhythm towards 24 h. In addition, the PRC also explains how, in non-equatorial zones, the sleep/wake cycle is aligned to the contracting and expanding dawn/dusk signal across the seasons. As illustrated in Figure 1A, the size of delaying phase shifts gets larger from subjective dusk into the night. So as night length gets shorter in the spring, delays will get bigger as more of the PRC is “exposed” to light. This delaying effect is counterbalanced by larger advances as more of the PRC is exposed to light as dawn gets earlier. In nature, entrainment arises from the averaging of delays at dusk and advances around dawn. In some nocturnal animals in northern latitudes, exposure to the long days of spring and summer can greatly compress night-time activity, but at least this activity will occur primarily in the dark and that time of day allowing the animal the best chances of survival. Although a direct comparison between a laboratory generated PRC and natural light exposure is not straightforward, the easiest way to think about the delaying and advancing impact of light on the circadian system is to consider a nocturnal mouse in the wild, emerging from its burrow during early dusk. Assuming it does not get eaten, the mouse will be exposed to light at a time that will delay its clock, and activity will start later the next day, with the mouse emerging after dusk and reducing the risk of predation. At the other end of the day, if the mouse has not retreated to its burrow at the end of the night, dawn light will advance its clock and activity will occur earlier the next day, giving the animal more time to complete its foraging before dawn arrives. In this way the activity pattern of the mouse is constantly being pushed back and forth so that it self-corrects around dawn and dusk. The situation is the same for diurnal species except that activity patterns must be located during the day. Again, dusk light will delay and dawn light will advance the clock, concentrating activity to the day and not the night.

In addition, light can act directly to modify behavior. In nocturnal rodents such as mice, light stimulates these animals to seek shelter, reduce activity and even sleep, whilst in diurnal species light promotes alertness and vigilance; e.g., Czeisler, et al. [11]. Therefore, circadian patterns of activity are not only entrained by dawn and dusk but also driven directly by light itself. This direct effect of light on activity has been called “masking,” and with the circadian system, restricts activity to that period of the light/dark cycle which is optimal for survival [12]. Across the animal kingdom, and especially the non-mammalian vertebrates, there is remarkable diversity in the light detecting (photoreceptor) mechanism whereby light is detected for circadian entrainment and masking [13,14,15,16]; the focus of this review will be confined to circadian entrainment in mice and humans.

## 2. The Discovery and Characterization of the 3rd Retinal Photoreceptor in Mice

**Figure 2:**
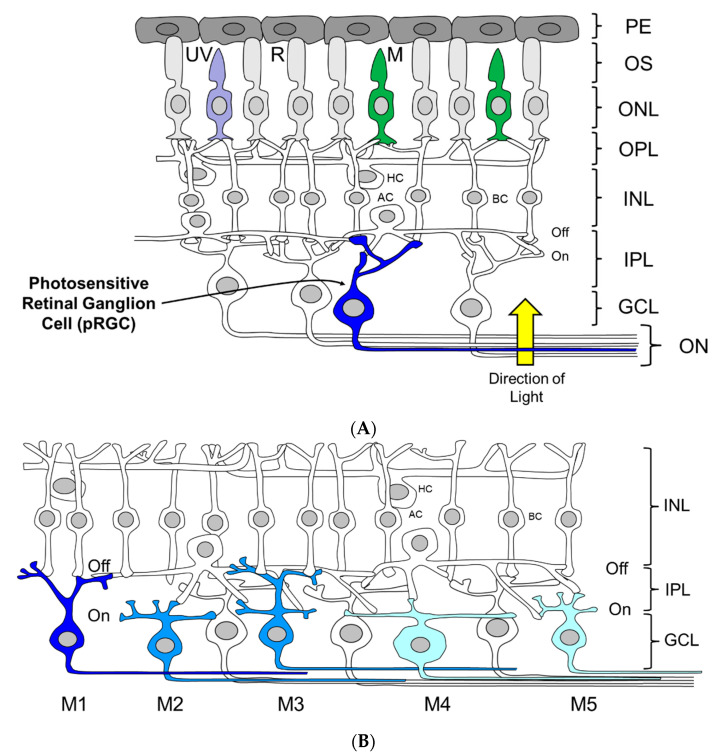
(**A**): Diagram of the Mouse Retina. The rods and cones have differing spectral maxima (λ_max_): rod photoreceptors (R) colored grey, λ_max_ ~ 498 nm; green cones (M) colored green, λ_max_ ~ 508 nm; ultraviolet sensitive cones (UVS) colored purple, λ_max_ ~ 360 nm. These photoreceptors convey visual information to the retinal ganglion cells via the second order neurons of the inner retina (INL), and the bipolar (BC), horizontal (HC) and amacrine (AC) cells. The optic nerve is formed from the axons of all the ganglion cells and this large nerve takes light information into the brain. A subset of photosensitive retinal ganglion cells (pRGC—shown in blue) detects light directly by using the “blue” light sensitive photopigment called melanopsin or OPN4. Thus, photodetection in the retina occurs in three types of cell: the rods, cones and pRGCs. The eye itself has an independent clock, which changes the sensitivity or the rods and cones to light, and to complicate matters still further, the pRGCs also receive signals from the rods and cones, via inner retinal neurons, and can help drive light responses by the pRGCs. Counter-intuitively, light passes to the rods, cones and pRGCs by passing through the inner to the outer retina. (**B**): A least five, and possibly six, subtypes of melanopsin-expressing pRGCs have been identified to date. Images showing the pRGC subtypes (1–5) identified in the mouse retina are based upon their intensity of labeling with melanopsin antibodies (indicated as dark to light blue) and their anatomy; specifically, their dendritic projections to the “ON” and “OFF” layers of the sublaminae of the inner plexiform layers (IPL). Most recently, a potential M6 cell has been identified which has a small bistratisfied dendritic field with spiny, highly branched dendrites (similar to M5 cells). Like other non-M1 pRGCs (including M4 cells), M6 cells project to the dorsal lateral geniculate nucleus, suggesting they contribute to pattern vision [16]. *Abbreviations*: inner nuclear layer (INL) which comprises multiple types of horizontal cells (H), bipolar cells (BC) and amacrine cells (AC); ganglion cell layer (GCL); optic nerve (ON); outer nuclear layer (ONL); outer plexiform layer (OPL); outer segments (OS); pigmented epithelium (PE); Off and On denote the ON and OFF sublaminae of the IPL.

Until relatively recently, the vertebrate’s eye had been considered thoroughly investigated, and viewed as perhaps the best understood part of the central nervous system. Years of painstaking research has explained how we see: Light is detected by the visual photoreceptors (rods and cones) which when stimulated produce graded electrical potentials. The inner retina then assembles these responses into a crude image. The retinal ganglion cells (RGCs) integrate this information, and via their axons which form the optic nerve, communicate with the brain, which then undertakes highly sophisticated visual processing in cortical and sub-cortical structures (Figure 2). Because visual responses could be broadly explained by the known physiology of the eye, the possibility of an additional ocular photoreceptor was never considered; in a sense, there was no need for such a proposition. Yet studies first in fish and then in rodents demonstrated that the rods and cones are not the only light sensing neurons of the vertebrate eye, and there exists another, entirely distinct class of ocular photoreceptor.

### 2.1. Identification of a 3rd Ocular Photoreceptor

The photosensitivity of rod and cone photoreceptors is based upon a photopigment which uses a vitamin-A-based chromophore called 11-cis-retinaldehyde embedded within a specialized protein termed an “opsin.” The opsin/vitamin-A photopigment is an integral membrane protein that possess seven trans-membrane-spanning domains. A photon of light is absorbed by 11-cis-retinaldehyde, which then undergoes photoisomerization to the all-trans state [17]. This 11-cis to all-trans conformation change alters the transmembrane helices which allows the opsin to interact with a G-protein signaling pathway that ultimately triggers a phototransduction cascade. Upon excitation, the rod and cone photoreceptors undergo a hyperpolarizing graded change in membrane potential that mirrors light intensity.

Much effort has been undertaken to define the rod and cone opsin genes of many species, and the visual opsins genes of teleost fish were thought to be fully characterized. As a result, the isolation of an additional opsin gene from the eye of the Atlantic salmon was surprising [18]. This new opsin gene family, discovered in 1997 and termed “vertebrate ancient” (VA) opsin, formed a fully functional photopigment and was shown to be expressed in a small number of retinal ganglion cells and horizontal cells, but was not expressed in the rods and cones [19]. The demonstration of non-rod, non-cone ocular photoreceptors generated surprise, if not incredulity, and many questions. Furthermore, this finding suggested that the growing body of evidence in mammals, that the retina might contain an unrecognized 3rd photoreceptor, should not be dismissed so quickly.

The discovery of an additional photoreceptor system within the retina of mammals came about as a result of trying to understand mammalian photoentrainment. Circadian clocks are not exactly 24 h and so must be entrained to the solar cycle to ensure that the internal and external day are appropriately aligned [20]. Publications from the early 1980s had shown that circadian and visual responses differ markedly in terms of the stimulus intensity and duration required to elicit a response; e.g., Foster and Helfrich-Forster [21]. For example, in the golden hamster, the threshold light intensity required for photoentrainment is more than 200 times greater than the intensities needed for the detection of a visual image, and requires stimulus durations of 30 s [22]. It needs to be stressed that photoentrainment in mammals relies exclusively upon ocular photoreceptors [23], and in this regard mammals differ markedly to the rest of the vertebrates which utilize multiple photoreceptors located within the pineal gland, hypothalamus and other areas of the brain [13,15,16]. Why mammals lost extraretinal photoreceptors is thought to be correlated with their early evolutionary history and what has been called a “nocturnal bottleneck” [24]. Ancestral mammals are all thought to have been exclusively nocturnal, and emerging from burrows at dusk would not have allowed sufficient exposure to light (intensity and duration) for reliable dawn/dusk detection by photoreceptors located within the brain. Thus, extraretinal photoreceptors were selected against, and only ocular photoreceptors persist in present-day mammals [25].

Because eye loss prevents photoentrainment in eutherian [23,26,27], and metatherian mammals [28], and because the visual photoreceptors were the only identified ocular photoreceptors, photoentrainment was attributed to these cells. This raised the question, “How can the rods and cones act as both image forming (IF) and non-image forming (NIF) dawn/dusk detectors?” [29].

In mammals, the master circadian pacemaker resides within the suprachiasmatic nuclei (SCN). Dawn/dusk information reaches the SCN from the retina via a monosynaptic projection called the retinohypothalamic tract (RHT) [30,31]. The RHT was identified in the early 1970s, but the specifics of the photoreceptor input remained poorly investigated. Early studies explored photoentrainment in mice with gene defects resulting in substantial loss of the rods and cones, including mice homozygous for the *rd/rd* mutation (*Pde6b^rd^*^1^) [32]. All rods are lost in the *rd/rd* retina, whilst approximately 5% of cone cells survive beyond 18 months, but in a highly degenerate state [27]. Despite the failure to respond to visual tasks, *rd/rd* mice display circadian responses to light that are indistinguishable from congenic mice with phenotypically normal retinas (*rd/+* and wildtype) [27,33]. Enucleation of these animals abolishes all circadian responses to light, showing that the photoreceptors must reside within the eye [27]. These reports in *rd/rd* mice differed from an earlier study suggesting that the *rd* (*Pde6b^rd^*^1^) mutation will attenuate circadian photosensitivity. In 1980 the threshold for entrainment in C57 wildtype mice was reported to be two log units more sensitive than C3H *rd/rd* mice (Table 1) [34]. The assumption was that the loss of classical photoreceptors (rods and cones) had attenuated circadian responses to light. However, the effects of genetic background on the *rd/rd* mutation, were not taken into account. C57 wildtype mice had been compared with C3H *rd/rd* mice. A later comparison of congenic C3H wildtype with C3H *rd/rd* mice showed that circadian photosensitivities were the same (Table 1). Differences in genetic background have also been a confounding factor in other studies. For example, the circadian photosensitivities of CBA/N (wildtype) and CBA/J (*rd/rd*) mice were compared, and CBA/J (*rd/rd*) mice were approximately 2 log units less sensitive than CBA/N (wildtype) mice [35,36]. Although mice were of the same strain, the interpretation of the results is again complicated because CBA/N mice were obtained from an inbred colony in Japan (Hamamatsu), whilst the CBA/J mice were obtained from a separate inbred colony from the USA (Jackson Laboratory). The mice were not of the same genetic background. Those results are discussed here because they illustrate the important point that even within the same species, or even the same strain, small genetic differences can give rise to altered thresholds for circadian entrainment [37,38].

The findings in *rd/rd* mice, and supported by studies on other rodent models, notably the blind mole rat (*Spalax ehrenbergi*) [40], suggested that the mammalian retina might contain an additional class of photoreceptor. Such a suggestion was initially dismissed on the basis that only a small number of rods and/or cones are required for normal photoentrainment, and a sufficient number of these visual cells were present within the degenerate mouse retina [41]. To settle this issue, mice were genetically engineered to lack all their rods and cones. This was achieved by crossing coneless transgenic (*cl*) mice [42] with either *rd/rd* mice [27] or transgenic mice (*rdta*) lacking rods [43]. Entirely normal photoentrainment of locomotor rhythms was observed in *rdta cl* mice [44], and *rd/rd cl* mice showed both normal circadian entrainment and the light suppression of pineal melatonin [45]. Enucleation blocked these responses, showing that the eyes must contain a novel photoreceptor. Collectively, these findings demonstrated that the mammalian retina, like that of teleost fish, must contain an additional class of photoreceptor. It also emerged that non-rod, non-cone photoreceptors are involved in a variety of other, non-circadian, light detecting tasks.

Pupil constriction is regulated by the rods and cones. However, it had long been noted that a robust light reflex of the pupil will still occur in animals with profound loss of the rods and cones, such as the Royal College of Surgeons (RCS) rat [46]. At the time it was assumed that the residual pupil light reflex was due to the survival of a small number of visual cells. The *rd/rd cl* mouse allowed an explicit test of this assumption, and the results showed these mice were fully able to constrict their pupils in response to bright light [47]. However, in contrast to circadian responses to light, there is a loss in sensitivity at low levels of light. This was the first suggestion that for some light detecting tasks there is likely to be a complex interaction between the classical and novel photoreceptors (see Section 2.8).

### 2.2. Identification of Photosensitive Retinal Ganglion Cells (pRGCs)

The hunt then began for the identification of the non-rod, non-cone photoreceptor. Two different approaches succeeded in identifying that a sub-set of retinal ganglion cells (RGCs) are endogenously photosensitive, and they have been called photosensitive retinal ganglion cells (pRGCs) (Figure 2A–B). Note–the terminology used for these cells in this review will be pRGCs. These cells are also variously referred to as melanopsin retinal ganglion cells (mRGCs) or as intrinsically photosensitive retinal ganglion cells (ipRGCs). One experimental approach involved injecting fluorescent microspheres into the SCN. These microspheres travelled through the axons of the RHT back to the retina and labeled RGCs. Recordings were then made from the labeled RGCs in the isolated retina bathed in a cocktail of drugs that largely blocks transmission of rod and cone signals to inner retina. Microsphere-labeled RGCs showed a light-dependent membrane depolarization which immediately suggested endogenous photosensitivity [48]. The drawback of this study was that it relied upon the effectiveness of the pharmacological blockade of inter-cellular communication. The second approach in mice used the isolated *rd/rd cl* retina combined with Ca^2+^ imaging techniques. The dye, FURA-2AM, fluoresces upon an increase in intracellular Ca^2+^ and was incorporated into the isolated *rd/rd cl* retina. Following light exposure, fluorescence imaging identified Ca^2+^ changes in approximately 3.0% of the RGCs (Figure 2A). Following the application of a gap junction blocker carbenoxolone, the number of RGCs responding to light dropped to approximately 1.0% of the RGC population, suggesting that the pRGCs are normally coupled via gap junctions to non-photosensitive RGCs. Furthermore, three different responses to light were identified in the pRGCs, characterized as sustained, transient and repetitive, suggesting that there might be different classes of pRGC [49]. As discussed below (see Section 2.6), the functional significance and mechanistic basis for these differences remains to be fully resolved.

### 2.3. Defining the Photopigment of the pRGCs Using Action Spectroscopy

**Figure 3:**
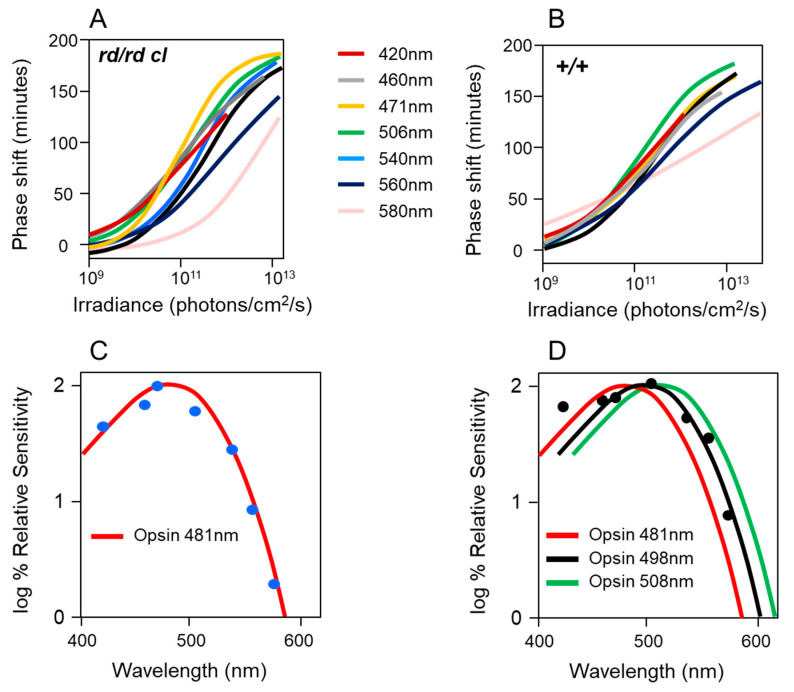
Action spectra for circadian entrainment. Action spectra were derived using the magnitude of the phase shift in a freerunning locomotor activity rhythm by a 15 min light stimulus. Wheel running activity rhythms of singly housed male C3H/He *rd/rd cl* and wildtype mice aged between 80 and 250 days were monitored. Animals were entrained to a L:D 12:12 cycle for seven days then placed under constant darkness. After 7 to 10 days of constant darkness, a single monochromatic (half band width = 10nm), 15 min light pulse of defined irradiance was applied four hours after activity onset (Circadian Time 16) to generate maximum phase delays. Animals were returned to constant darkness for a further 10 days. The magnitude of the phase shift was calculated by comparing the time of activity onset before and after the light pulse. Pre-pulse phase was calculated from the seven days prior to the light pulse application, and the post-pulse stable freerunning activity was calculated from the seven days after the light pulse, taken from the second day after the light pulse. Monochromatic and neutral density filters were used to regulate the wavelength and intensity of the stimulus allowing irradiance response curves to be compiled at 420, 460, 471, 506, 540, 560 and 580 nm. Irradiance response curves (IRCs) were compiled at seven wavelengths of near-monochromatic light (n = 4 to 7 animals for each data point) between 420 and 580 nm in: (**A**) the *rd/rd cl* and (**B**) wildtype mice. (**C**) The derived action spectrum for circadian entrainment in *rd/rd cl* is well approximated an opsin-retinal photopigment with a novel λ_max_ at 481 nm (R-squared = 0.976). (**D**) The wildtype action spectrum is also well approximated by an opsin-vitamin A photopigment (R-squared = 0.896), but with a λmax of ~500 nm. This is consistent with the involvement of a rod (498 nm) and/or cone (508 nm) absorption spectra.

The opsin/vitamin A-based photopigments of the animal kingdom show a remarkable diversity in their wavelengths of maximum sensitivity (λ_max_), absorbing maximally from the ultraviolet (UV) to the far-red/infra-red part of the spectrum. Despite this range, all have a characteristic absorption profile similar to a bell-shaped curve and called the opsin/vitamin-A photopigment nomogram. This means that an “action spectrum,” which describes the spectral sensitivity of a light-dependent response, can be genera to identify a particular photopigment type. Generating an action spectrum can be complex and time consuming as it requires the construction of full dose response curves across a range of monochromatic light stimuli [50,51]. Action spectroscopy was used to try and identify the photopigment of the pRGCs, and the first constructed was for pupil constriction in *rd/rd cl* mice. The action spectrum demonstrated that the photosensitivity of the pRGCs is based upon an opsin/vitamin A-based photopigment with a λ_max_ at 479 nm. This photopigment was tentatively named OP479 (opsin photopigment λ_max_ 479 nm) [47]. An additional action spectrum was then made for circadian entrainment (Figure 3), and it identified an opsin/vitamin A-based photopigment with a λ_max_ at 481 nm [52], so one highly similar to that for pupil constriction (λ_max_ 479 nm) [47]. These results suggested that the same photopigment regulates both pupillary and circadian responses to light. In contrast to the *rd/rd cl* results, the action spectrum from congenic wild-type mice best fits an opsin-vitamin A photopigment with a λ_max_ of ~500 nm. These data suggested that normally, photoentrainment is in some way influenced by an input from the rod (λ_max_ 498 nm) and/or cone (λ_max_ 508 nm) photoreceptors. This will be discussed in detail below (see Section 2.8).

**Figure 4:**
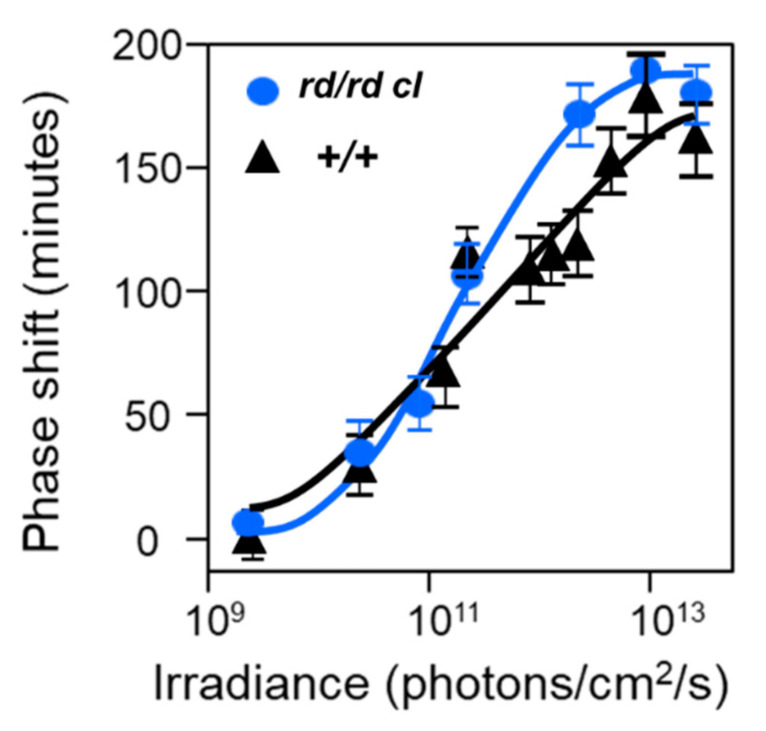
A comparison of *rd/rd cl* and wildtype responses at 471 nm (15 min exposure) The data show a similar irradiance range of responses in the two genotypes, from ~1 × 10^9^ photons cm^2^ s^−1^ to a saturating response at ~1 × 10^14^ photons cm^2^ s^−1^. This dynamic range corresponds to both the rod and cone activation ranges. A significant difference is identified in the slopes of the response relationship of the irradiance response curves (*p* < 0.002), and photobiology formalisms suggest that this represents responses driven by different photoreceptors in the two genotypes.

The action spectrum for circadian phase shifts in the *rd/rd cl* mouse provided a good fit to an opsin-retinal template with a novel λ_max_ at 481 nm (Figure 3C). When retinal is bound to an opsin as a photopigment, the λ_max_ is dependent on and specific to the opsin protein. The shape of the *rd/rd cl* action spectrum strongly suggested that the non-rod, non-cone photopigment is based upon an opsin/retinal photopigment with a λ_max_ of 481 nm. However, this λ_max_ does not correspond to the defined mouse rod and cone photopigments (Figure 2A), and so suggested a novel photopigment class. Similarly, the wildtype action spectrum does correlate well with a single M cone or rod λ_max_ and suggested that both the rods and cones provide light information to the circadian clock. Further support for the involvement of multiple photopigments in the wildtype response was provided by a detailed comparison of the irradiance response curves (IRCs) of *rd/rd cl* and wildtype mice at 471 nm (Figure 4). A significant difference in the slope of the response was identified between the genotypes and this again suggested the involvement of different or additional photoreceptors in the wildtype response. It is also important to note that despite the loss of rods and cones in *rd/rd cl* mice, a similar irradiance response range was apparent, and the irradiance required to generate a 50% response was the same for both genotypes. This demonstrates that although the rods and cones might contribute to phase shifting responses, the pRGCs can operate effectively on their own across the dynamic range of wildtype responses; indeed, a recent report suggests that pRGCs might be capable of detecting light at levels much dimmer than previously expected [53]. One possibility for the broad sensitivity range of pRGCs is that different pRGC populations, with different and overlapping sensitivity ranges, combine to provide sensitivity across an extended range of irradiances [54].

**Figure 5:**
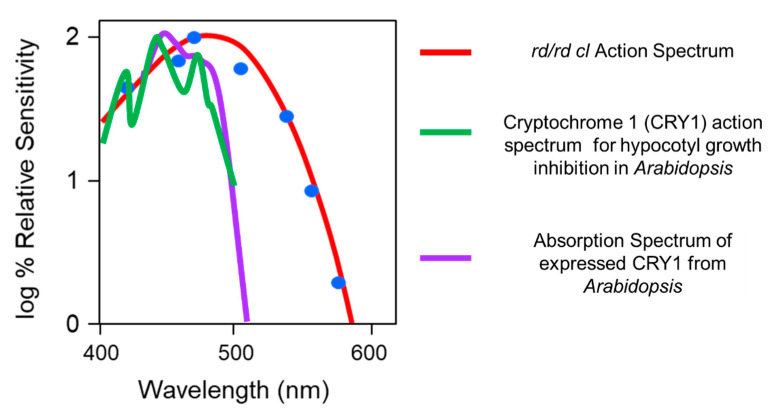
Comparison of flavin-photopigment-based and opsin/vitamin A-based spectral responses. The well-defined action spectra for flavin-photopigment-based responses correspond closely to each other but not to the action spectrum for circadian phase shifts in *rd/rd cl* mice. See text for details.

The action spectrum for phase shifting in the *rd/rd cl* mouse also addressed the widely held belief by some that cryptochrome (CRY) might act as a photopigment for photoentrainment [55,56,57]. For a review, see [58]. As there were no absorption spectra for mammalian cryptochromes, the *rd/rd cl* action spectrum was compared to a detailed action spectrum that existed for a flavin-based photopigment [59] (Figure 5). Although the plant CRYs absorb in the blue part of the spectrum, there was no match between the Flavin-based CRY1 photopigment of *Arabidopsis* and the action spectrum for phase shifting in the *rd/rd cl* mouse, which fits an opsin-retinal absorption spectrum nomogram.

Action spectra have also been determined by direct recording from pRGCs in rats [48] and the macaque [60]. These and additional studies have shown that pRGCs utilize an opsin photopigment with a λ_max_ very close to 480 nm. In this regard the λ_max_ of the pRGCs seems to be a highly conserved feature, unlike the cone opsins in these species, and there has been debate regarding why, and what ecological advantage that this λ_max_ might confer. An attractive answer is that the pRGCs are “spectrally tuned” to the dominant wavelength of light during twilight. When the sun is close to the horizon the light at the horizon is enriched with red light, but the dome of the sky is dominated by “blue” light near 480 nm. This is because there is a strong wavelength-dependent scattering (~λ^−4^) of light by particles in the atmosphere, such that shorter (blue) wavelengths are scattered more strongly than longer (red) wavelengths. The result is that indirect blue light dominates the dome of the sky [21].

### 2.4. The Identification of Melanopsin (OPN4)

**Figure 6:**
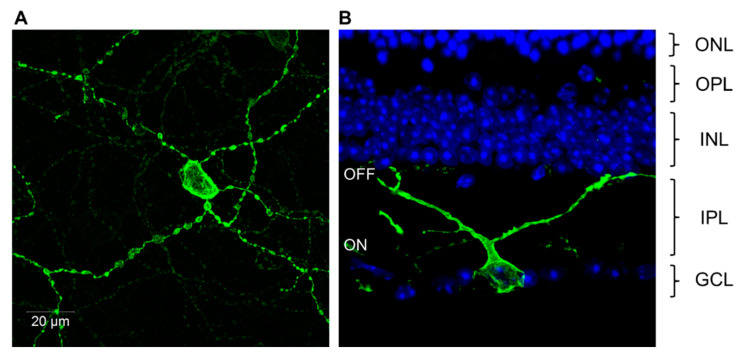
Visualization of melanopsin expressing photosensitive retinal ganglion cells (pRGCs) of the adult mouse retina. (**A**) Confocal microscopy image of a flat mounted mouse retina showing antibody labeling of melanopsin in an M1 type pRGC with high levels of melanopsin expression and large but sparse dendritic fields. Additionally, note the presence of a weaker stained processes from neighboring non-M1 cells. (**B**) Cross section image of the mouse retina showing the dendrites of M1 type pRGCs extending to the OFF layers of the inner plexiform layers (IPL). *Abbreviations*: Outer nuclear layer (ONL); inner nuclear layer (INL); inner plexiform layer (IPL); ON and OFF mark the ON and OFF sublaminae of the IPL; ganglion cell layer (GCL).

Action spectroscopy in *rd/rd cl* mice had defined the biochemistry of the photopigment employed by the pRGCs, but the genetic identity of the opsin remained unresolved. One possibility was that there would be a mammalian ortholog of teleost VA-opsin, and that mammals and fish would share similar inner retinal photopigments. However, no VA opsin genes were identified in mammals, and the answer rested in another newly discovered family of opsin genes, called the melanopsins (*Opn4*). The first *Opn*4 gene was isolated from the light sensitive pigment cells (melanophores) of the African clawed toad *Xenopus* [61], and was then found in other vertebrate species, including several teleost fish [62], and multiple mammalian species, including humans, mice, cats and the marsupial or fat-tailed dunnart [63,64,65]. A key finding was that that melanopsin (*Opn*4) was expressed within a small population of RGCs. In addition, the anatomy and distribution of the melanopsin expressing RGCs was highly similar to the RGCs that formed the RHT [66]. Examples of melanopsin expressing pRGCs, identified using antibodies raised against mouse melanopsin, are shown in Figure 6.

The link between melanopsin and the capacity of the pRGCs to respond to light was provided by the genetic ablation of *Opn4*. Mice lacking functional melanopsin (*Opn4*^-/-^) lost direct photosensitivity of the pRGCs, showed attenuated photoentrainment and exhibited only a partial pupil constriction [67,68,69]. In addition, when mice lacking functional rods and cones were crossed with *Opn4*^-/-^ mice, all responses to light were lost [52,70]. Thus rods, cones and melanopsin-based pRGCs undertake all known light detection by the eye. The triple knockout results answered another question. Cryptochrome (CRY) was being strongly promoted as the circadian photopigment of mammals [56,57,71], and despite the lack of evidence (see discussion above and Figure 5), the loss of circadian responses to light in mice lacking rods, cones and OPN4 finally excluded the possibility for a CRY-based photoentrainment mechanism. The current consensus is that the CRYs do not form photopigments in the mammals. For additional discussion, see [51].

### 2.5. Melanopsin Expression Studies

Although the gene ablation studies demonstrated that OPN4 is a required for pRGC photosensitivity, this approach alone cannot formally demonstrate that OPN4 is a photopigment [17]. Whether melanopsin can form a fully functional photopigment was initially addressed by expressing OPN4 in COS cells and demonstrating that melanopsin expressing COS cells can mediate light-dependent G-protein activation [72]. This was followed by three groups who expressed either human or mouse melanopsin in Neuro2A cells [73], HEK293 cells [74] and *Xenopus* oocytes [75]. Significantly, all showed that OPN4 could initiate a retinal (chromophore)-dependent signaling cascade in response to light. For example, expression of OPN4 in neuroblastoma (Neuro2A) cells, and following the addition of retinal chromophore (9-*cis*-retinal or 11-*cis*-retinal) to the culture media, transformed Neuro2A cells into functional photoreceptors. An additional finding was that melanopsin is able to regenerate its chromophore in response to light, converting all-*trans*-retinal back to its functional 11-*cis*-retinal state. Thus, melanopsin is a “bi-stable photopigment” and in this regard similar to the invertebrate photopigments [73]. These findings were subsequently replicated several years later [76,77]. Bi-stability has also been observed in the melanopsins of non-mammals, including the cephalochordate amphioxus [78], suggesting that this is a conserved feature across all chordate melanopsins. This might be an important adaptation, allowing melanopsin-based photoreception in cells which are not adjacent to cells/tissues involved in chromophore recycling (11-*cis*-retinal ⇔ All-*trans*-retinal), such as the retinal pigment epithelium [73].

### 2.6. OPN4 and pRGC Complexity

Since their identification, pRGCs have been shown to contribute to a broad range of non-image forming (NIF) responses to light [29], including: pupillary light response (PLR) [47]; the acute suppression of locomotor activity (negative masking) [79]; sleep induction [80,81,82,83]; levels of alertness [84,85,86]; light aversion [87,88]; and influencing mood-related behaviors, such as levels of anxiety and cognitive function [85,86,89]. More recently it has been discovered that melanopsin contributes not only to NIF responses to light but also visual pathways, challenging the previous model of separate image forming (IF) and non-image forming (NIF) systems [90,91,92]. For example, melanopsin-based pRGCs convey light to the visual centers of the brain regarding overall levels of environmental light, and perform roles in irradiance coding and brightness discrimination [93,94,95,96], contrast detection [97] and adaptation of visual responses [98], whilst also possibly providing spatial information and potentially supporting basic pattern vision [99,100].

Melanopsin also acts as an irradiance detector at the level of the retina [98,101] and facilitates the adaptation of cone photoresponses to bright light [98,102,103]. Intraretinal signaling from pRGCs has been shown to influence the activity of dopaminergic amacrine cells [104,105,106] and displaced (AII) amacrine cells [107], and regulate the spike firing rate of large numbers of (non-melanopsin) retinal ganglion cells to control the rate of information transmission through the optic nerve [108]. During postnatal development, melanopsin regulates the calcium waves that spread across the retina and promotes the segregation and refinement of retinofugal projections [109,110,111]. Such findings demonstrate that the melanopsin system is far more complex than first appreciated, contributing to a wide variety of physiological and behavioral responses. How melanopsin-based pRGCs are capable of driving so many responses remains unclear, but it must be related in some way to their functional diversity.

The pRGCs do not form a uniform population of cells. Instead they constitute an anatomically, genetically and functionally distinct set of subtypes (Figure 2B). At least five and possibly six pRGC subtypes have been identified, termed M1–M5 [50,99,112,113,114]. They differ in their morphology and retinal connections, and show light responses with distinct properties [92,115,116]). In addition, pRGC subtypes seem to project to different brain regions [99,112,113,117,118,119], and as a result, may mediate different physiological responses to light [114,115]. However, and as discussed below, some caution needs to be exercised linking specific pRGC sub-types with specific physiological and behavioral functions; it is not straightforward, as the discussion below will illustrate.

The first pRGC sub-type to be identified was the M1 pRGC (Figure 2B). These have high levels of melanopsin expression and show stratification of their relatively sparse dendrites exclusively within the OFF sublamina of the inner plexiform layer (IPL) [112,120]. M2 pRGCs sub-types were then identified, followed by M3 cells. M2 pRGCs express lower levels of melanopsin compared to M1 cells, and have more complex dendritic fields which are restricted to the ON sublamina of the IPL [112,113,114,121]. By comparison, M3 pRGCs are bistratified with dendrites present in both the ON and OFF layers of the IPL [115,121] and show an intermediate level of melanopsin expression [121]. Further subtypes of pRGC were subsequently identified termed M4 and M5, largely as a result of developing transgenic *Opn*4.Cre based mouse lines utilizing a range of fluorescent reporters [99,122,123], which allowed the visualization of additional cell types expressing levels of melanopsin that were too low to be reliably detected using OPN4 antibodies [99,121,122]. The anatomy of M4 and M5 cells is broadly similar to that of M2 type cells, having dendrites confined to the ON layer of the IPL. However, these cell types are distinguished based upon low levels of melanopsin expression and the size and complexity of their dendritic fields, and in the case of M4 cells their large cell bodies [99], and the expression of the neurofilament heavy chain protein SMI-32 [124]. Most recently, the number of pRGC subtypes in the mouse may have been extended further with the discovery of the M6 type pRGC [50]. M6 cells are bistratified (similar to M3 cells) with small, spiny and highly branched dendritic fields (similar to M5 cells). In addition to these anatomical classifications, single cell transcriptome approaches have also been used to study the diversity of pRGCs, and have again identified a number of subpopulations defined by characteristic profiles of gene expression. However, these groups do not perfectly correspond to the anatomical subtypes currently described (i.e., M1–M5 cells) indicating further complexity in pRGC diversity [125].

The projections of M1 type pRGCs into the brain have been well characterized, using *Opn*4.tau.LacZ mice that selectively label M1 pRGCs [112]. M1 pRGCs project to a number of brain regions associated with NIF functions, including (but not limited to) the suprachiasmatic nucleus (SCN), intergeniculate leaflet (IGL), olivary pretectal nucleus (OPN), ventrolateral preoptic area (VLPO), ventral lateral geniculate nucleus (vLGN), medial amygdala, peri-habenula and superior colliculus (SC) [86,112]. More recent studies using *Opn*4.Cre mice have identified the projections from all the pRGC subtypes (M1–M5) [99,117], and identified a number of additional brain targets, including the dorsal lateral geniculate nucleus (dLGN), the OPN (core region) and the superior colliculus (SC) [99]. However, it is important to note that these *Opn*4.Cre models do not allow the identification of projections from individual pRGC subtypes; rather, they label the combined innervations of all pRGCs. As a result, the brain regions innervated specifically by M2–M5 type pRGCs remain largely undetermined. However, retrograde labeling from the SCN and OPN has shown that the SCN is innervated primarily by M1 type pRGCs (80%) with a lower projection from M2 type cells (20%) [113]. In contrast, the OPN receives an input from both M1 (55%, mainly shell region) and M2 cells (45%, mainly core region) [113]. Additional studies have shown that the dLGN is innervated primarily by M4 type pRGCs, but also receives input from other pRGCs, but not M1 pRGCs [119]. Finally, the putative M6 cells also project to the dorsal lateral geniculate nucleus [50]. Interestingly the SC would seem to be widely innervated by pRGC subtypes, receiving projections from M1–M5 cells [126].

On the basis of their anatomical projections, it would not be unreasonable to propose that different pRGC subtypes mediate distinct responses to light [115]. However, in most cases the specific roles of each pRGC sub-type remain unclear. The most notable exception would be the role of M1 cells in photoentrainment and the PLR. The first point is that the M1 type pRGCs that innervate the SCN and OPN comprise two molecularly distinct subtypes of M1 pRGCs, distinguished by their differential expression of the transcription factor Brn3b [118]. Remarkably, a sub-population of approximately 200 SCN projecting M1 pRGCs (Brn3b negative) are capable of driving circadian entrainment following the genetic ablation of almost all other pRGCs (Brn3b positive, M1-M5 pRGCs) [118]. The ablation of all Brn3b positive pRGCs was, however, shown to disrupt the pupillary light response (PLR). Thus, distinct subsets of M1-type pRGCs appear to drive circadian entrainment and the PLR.

Currently, the functions of the other classes of pRGCs remain poorly defined, and information is lacking regarding the responses driven by M2, M3 or M5 type pRGCs. However, some information does exist regarding the role of M4 type pRGCs. The nature of the light responses recorded from the dLGN suggests a role for melanopsin in encoding background illumination [94,95,96] and in driving the adaptation of visual responses to permit the encoding of complex visual signals [98,101]. Significantly, retrograde labeling studies have shown that M4 type pRGCs project almost exclusively to the dLGN [119], and so these pRGCs seem to be primarily tasked with modulating pattern forming vision. However, the dLGN also receives projections from other non-M1 type pRGCs [119], including the recently described putative M6 pRGC [50], and as a result it remains difficult to define the specific contribution of M4 cells (or other classes of non-M1 pRGCs) to the integration of light information by the dLGN.

### 2.7. Diversity of Melanopsin Light Responses

The first Ca^2+^ imaging studies on pRGCs noted that multiple types of light response (repetitive, sustained and transient) can be recorded from the pRGCs of the mouse retina [49]. A similar diversity was also identified using multiple electrode array (MEA) recordings: type I (found only in neonates, strongly light sensitive with slow onsets and fast offsets); type II (found in adults, relatively insensitive to light with slow onsets and slow offsets); and type III (found in adults, strongly sensitive to light with rapid onsets and very slow offsets) [127]. Very recently, a similar response range has been shown in human pRGCs [128]. The straightforward hypothesis would be that, as the type of light information required to drive different behaviors, such as circadian photoentrainment (integrating over time) and the PLR (rapid and transient response), are likely not the same, the pRGCs that innervate each distinct brain area will exhibit different functional properties in order to meet these demands. Frustratingly, the precise relationships between the different light responses and specific pRGC subtypes (Figure 2B) remain only poorly resolved, and will be important topics for future studies. Below we attempt to tease apart what we do know about the light responses of pRGCs.

Perhaps the first point to make is that in addition to their endogenous melanopsin-based light responses, all pRGC subtypes (like conventional RGCs) receive indirect inputs from the rods and cones [60,99,114,122,129,130,131,132,133,134]. As a result, trying to assess how pRGCs respond to light in the natural world is complicated. Originally, M2 cells were thought to receive greater levels of excitatory input from rods and cones compared to M1 cells [88], suggesting that the rods and cones provide greater modulation of M2 cells compared to M1 cells [133]. More recently, however, studies have suggested that all pRGC subtypes (M1–M5) receive similar levels of excitatory input from rods and cones [129]. Rod and cone inputs to the pRGCs are not direct, and despite differences in their patterns of stratification (Figure 2B), the ON pathway forms the dominant excitatory synaptic input to M1–M5 type pRGCs [114,126,130,135,136]. This input from the ON pathway is derived from both rods and cones, at least in the mouse retina [129,137]. M1 type pRGCs also receive low levels of excitatory and inhibitory input from the OFF pathway of the retina [126,130]. Intracellular recording from the macaque retina has shown that melanopsin pRGCs are inhibited by the short-wavelength cones (S cones), whilst the rods and medium-wavelength cones provide an excitatory input [60]. By contrast, recent studies in humans have failed to find evidence for an S cone contribution to acute neuroendocrine and alerting responses to light [138]. Finally, multiple lines of evidence from behavioral studies have implicated an input from the rods and cones [40,139,140,141], not least the finding that *Opn*4^-/-^ (knockout) mice still show circadian entrainment, albeit in an attenuated form [67,68,69]. Before continuing the discussion of the endogenous responses of the pRGCs, we make the point again that in the natural world, the outputs from the pRGCs will be the product of their endogenous photosensitivity, the input from other connected pRGCs and a potentially very important input from the rods and cones.

The original studies on M4 and M5 pRGCs suggested that these cells have only small endogenous responses to light, and this would be consistent with their very low levels of melanopsin expression [99] (Figure 2B). However, subsequent studies have reported that the melanopsin-driven light responses of M4 and M5 pRGCs are similar in sensitivity and amplitude to those of M2 type cells [126]. It is worth noting that despite their significantly larger photocurrents, M1 pRGCs exhibit maximal spike firing rates that are significantly lower than those of M2–M5 type pRGCs [126,142], an observation that might be explained by the increased tendency of M1 cells to enter into a state of depolarization block (a block in the generation of action potentials) during light responses [99,114,130]. However, rather than being a sign of excitotoxicity, this feature could represent a functional specialization of the M1 subtype related to their in role in circadian photoentrainment and the tuning of individual M1 pRGCs to specific intensities of light [54].

Most recently, detailed patch clamp studies of defined M1–M5 pRGCs indicate that the light responses recorded from pRGCs of the adult mouse retina can be broadly divided into M1 and non-M1 type responses with the responses of M1 cells being “light sensitive, small in amplitude, with a fast onset”; and the responses of M2–M5 cells are similar to each other, and are “less sensitive to light, large in amplitude, with a slow onset” [126,142]. Based on these properties, there is now a general consensus that M1 cells correspond to the originally described type III responses (sensitive with rapid onset and very slow offset) [127], and that M2–M5 type cells combined represent the type II responders (insensitive with slow onset and slow offset) [127]. Interestingly, M4 cells express much higher levels of melanopsin during postnatal development, and at these time points produce light responses typical of type I responses [143]. The marked loss of melanopsin expression within M4 cells during development explains the loss of type I responses in the adult mouse retina [143]. It should be noted, however, that there is significant heterogeneity within each class of light response, such that type II responders and type III responders can both show sustained (slow offset) and persistent (very slow offset) responses [143]. Significant differences in light responses are also observed between pRGCs of the same subtypes [144,145], with surprisingly large biophysical diversity reported for M1 pRGCs [54,146]. As suggested earlier, it is tempting to attribute this diversity of M1 responses to the different subpopulations of M1 cells innervating different brain regions, but attempts to record and compare responses from specific sub-populations of M1 pRGCs (identified by retrograde labeling) have failed to identify differences between the M1 pRGCs projecting to the SCN and OPN, with both populations showing similarly diverse ranges of response properties [146]. Consequently, the significant diversity in M1 photoresponses cannot be explained either by the sites they project to, or the light-sensing tasks they mediate. Both the SCN and OPN receive an equally diverse set of inputs. Furthermore, different light responses can be generated within the same pRGC depending on the activation of cAMP second messenger systems [147], levels of dopamine signaling [148], light history and the wavelength of light [77]. Responses of pRGCs may also be spectrally tuned depending on their location in the retina [122]. It is becoming clear that individual pRGCs are capable of generating responses under different exogenous and endogenous environmental conditions. Thus, melanopsin-based light detection is anything but simple!

It is also important to note that the properties of pRGC light responses are often measured under specific conditions that may not relate to normal physiological conditions, often involving short pulses (typically 1–10 s) of monochromatic light under dark adapted conditions. Furthermore, studies have tended to focus either on the endogenous melanopsin driven responses of these cells (following chemical blockade of rod and cone signals, or early postnatal tissue for example), or alternatively, on characterizing the nature of rod and cone inputs to pRGCs in the absence of melanopsin driven signals. By comparison, the combined rod, cone and melanopsin responses of pRGCs (the actual output of pRGCs) have received surprisingly less attention, and when reported, are typically not performed under physiologically relevant light paradigms. It therefore remains to be resolved how distinct subtypes of pRGCs integrate rod/cone and melanopsin signals under “natural” environmental lighting conditions and how the true repertoire of pRGC signaling responses (and functional outputs) may vary. It is only when we can truly understand this that we will be able start explaining how the properties of pRGC light responses may be specialized for the roles they perform.

Despite the limitations in our understanding, based upon the observations outline above, it is clear that melanopsin signaling is a diverse and dynamic phenomenon, resulting in cellular light responses (and outputs) with markedly different kinetics. While the physiological relevance of such diversity remains unclear, it is also evident that the cellular basis for generating such a variety of pRGC response is also poorly understood. The mechanisms of phototransduction in melanopsin expressing pRGCs are known to involve a membrane bound signaling cascade involving Gq/11 type G-proteins, activation of PLCβ4 and ultimately the opening of downstream TRPC6 and TRPC7 ion channels [149,150,151,152,153,154,155]. However, this model only describes the basic core components of what is likely to be a far more complicated signaling pathway, and such a simple model fails to account for the diversity of pRGC light responses observed. Data have been largely obtained from M1 type pRGCs, and it is currently unclear whether the mechanisms of melanopsin phototransduction are conserved between different pRGC subtypes. Recent studies have begun to clarify this issue and fundamental differences have been observed in the downstream signaling cascade employed by M1 (classically circadian) and M4 pRGCs (dLGN, proposed role in vision) [156]. Contradicting the basic model of pRGC phototransduction, changes in cellular excitability and spike firing of M4 cells are driven by PLC dependent closure of background leak ion channels, likely TASK type channels of the K2P family of potassium channels [156], and not opening of TRPC-type cation channels as reported for M1 cells. Closure of background leak K^+^ channels depolarizes the resting membrane potential and enhances the cellular excitability of M4 cells, resulting in enhanced contrast sensitivity following tonic exposure to even relatively dim background light, consistent with their presumed role in pattern vision [156]. This study clearly indicates that melanopsin phototransduction is not a fixed constant but instead is repurposed within different pRGC subtypes in order to reshape the properties of cellular light responses. Again, the detailed mechanisms of melanopsin phototransduction, and how they vary between pRGC subpopulations and also under different physiological conditions, remain unresolved.

The potential for further complexity in the melanopsin phototransduction also arises because there are two distinct isoforms of melanopsin. These isoforms are generated by alternative splicing of the single melanopsin gene [157]. This results in a short (OPN4S) and a long (OPN4L) form of melanopsin protein with differences in their C-terminal tails. Bioinformatic analysis suggests that the longer tail of OPN4L contains more phosphorylation sites [157], and that this may result in functional differences between the two proteins, most likely influencing rates of adaptation, de-activation and recovery following light exposure [158,159,160,161,162]. Whilst this seems likely, the key residues regulating β-arrestin binding and melanopsin de-activation are seemingly conserved between OPN4L and OPN4S [163]. Notably, OPN4S and OPN4L are found in different subtypes of pRGC at different levels of expression. M1 and M3 type pRGCs express both OPN4S and OPN4L, whereas only OPN4L is detected within M2 type cells [157,164]. Unfortunately, due to the low levels of melanopsin expressed with M4 and M5 type cells, it has not been possible to determine which isoforms are expressed within these cells.

Clearly, different levels of the two melanopsin isoforms may provide the substrate for generating different response profiles within the M1–M5 subtypes, and there is evidence to support this. Silencing of OPN4L and/or OPN4S expression in vivo has been shown to produce different effects upon a range of NIF responses [165]. The silencing of OPN4S alone was sufficient to disrupt the PLR, whilst silencing both OPN4S and OPN4L was necessary to greatly attenuate the phase-shifting of locomotor behavior and the induction of sleep. By contrast, negative masking (the suppression of locomotor activity) was attenuated by silencing of only OPN4L, with no apparent dependence on OPN4S. Based upon these observations, it seems probable that OPN4S and OPN4L, expressed at different levels within different pPRG sub-types, and driving different signaling pathways, may be in part responsible for driving different behavioral responses to light [165].

### 2.8. Rod, Cone, pRGC Interactions at the Level of the SCN

The results from *rd/rd cl* mice demonstrated that rods and cones are not required for photoentrainment [44]. However, we did not conclude that the rods and cones play no role (see Figure 3). Indeed, the discussion above highlighted the fact that different classes of pRGCs appear to receive different inputs from the rods and cones [60,99,114,122,129,130,131,132,133,134]. Several important studies have explored in detail whether the rods and cones of the mouse retina contribute to the light information received by the SCN and whether this information is used for circadian entrainment and other NIF responses to light. Electrophysiological recordings from the SCN of unanesthetized and freely moving mice show that the SCN increases its electrical activity when mice are exposed to UV light, a stimulus that would maximally stimulate the UV cones (Figure 2A). The response is characterized by fast-transient components occurring at the light transitions and sustained spike firing that depends upon the level of illumination [166]. In parallel with SCN recordings, circadian phase-shifting of locomotor behavior and light-induced sleep induction can be driven by UV light. Both the UV-induced electrical responses from the SCN and the behavioral responses were maintained in mice lacking melanopsin (*Opn4*^-/-^), or functional rod photoreceptors (*rd/rd*), but greatly attenuated in mice lacking both rods and cones (*rd/rd cl*). The residual UV sensitivity in *rd/rd cl* mice is explained by the alpha absorption spectrum of melanopsin which overlaps with the UV part of the spectrum. These findings provided very strong evidence that UV responses to light in mice are mediated by UV cones (Figure 2A) [166].

In response to retinal illumination, SCN neurons show an increase in spike frequency [76,167,168,169,170]. That consists of two components, a fast transient at the onset and offset of the light signal, with sustained/tonic firing, where spike frequency is dependent upon light intensity [171,172]. These distinct responses have been thought to arise from specific rod/cone and melanopsin inputs, with sustained responses originating in melanopsin pRGCs and the transients from the rods/cones [76,170,173]. However, studies on wildtype mice, mice lacking melanopsin (*Opn4*^-/-^) and mice lacking rods and cones (*rd/rd cl*) suggest that this may not be the case. Electrical activity recordings from the SCN of freely moving mice showed an acute irradiance-dependent firing of SCN neurons upon UV (λ_max_ 365 nm), blue (λ_max_ 467 nm) and green (λ_max_ 505 nm) light exposure. These responses were sustained for the full duration of the stimulus. Unexpectedly, the sustained/tonic response was unaffected by the loss of melanopsin, but was strongly attenuated by the loss of rods and cones! Furthermore, melanopsin can mediate both sustained and fast transient (on/off) responses to light in the absence of the rods and cones. These results showed that classical photoreceptors play an important role in transmitting irradiance information to the central pacemaker of the mouse SCN [174], and that melanopsin pRGCs can encode both transient and sustained responses to light.

An additional approach to address the contribution of cone photoreceptors in circadian entrainment has been to provide stimuli of a short duration over an extended time period with high temporal contrast. The work of Nelson and Takahashi [22] had explored the action of light as a synchronizer on the circadian system in the golden hamster (*Mesocricetus auratus*) and showed that the circadian system is capable of integrating photons over tens of minutes, allowing discontinuous stimuli to be assimilated and used to evoke phase shifts—an ability that would be useful when moving around within an environment with intermittent shade (Table 2). More recent studies have taken advantage of this feature of the circadian system by presenting a total illumination time of 15 min as a series of 1 min pulses spread over 43 min; i.e., each 1 min pulse separated by 2 min of darkness. This protocol drove phase shifts of equivalent magnitude to a continuous 15 min pulse in *rd/rd cl* mice. These researchers then used a mouse with a red cone knock-in allele (referred to as *Opn1mw^R^*) which results in a substantial, long-wavelength shift in the spectral sensitivity of the M cones (Figure 2A) from 508 nm to approximately 560 nm. In these mice, responses to the continuous and discontinuous light stimuli at 500 nm were indistinguishable. By contrast, discontinuous light stimuli at 644 nm greatly enhanced phase shifting responses to light in *Opn1mw^R^* mice. Because the 644 nm light would preferentially stimulate the 560 nm cones in *Opn1mw^R^* mice, the conclusion is that high temporal contrast, detected by cones, provides a significant additional input to the SCN. These findings are supported by earlier studies showing that intermittent light exposure, presumably detected by the cones, provides an important signal to the SCN [22,175,176].

In summary, studies using a range of approaches have demonstrated an important contribution of rod and cone photoreceptors in photoentrainment. Although the following statement is likely to be an oversimplification, rods seem to usually contribute to photoentrainment at low light levels; cones transduce light information at intermediate and high irradiances and are able to integrate intermittent changes in light levels; melanopsin pRGCs detect higher irradiance light and integrate light information over extended periods of time.

### 2.9. The Intensity, Duration and Spectrum of Effective Light Stimuli—Ecological Relevance

The discussion in the sections above have highlighted the fact that the light inputs to the circadian system of non-human species are immensely complicated involving a diversity of photoreceptors (pRGCs 1–5; rods and cones) and signaling pathways. Why there is this complexity, and the precise mechanisms whereby these photoreceptor systems interact, remain unclear. However, at a fundamental level, answering these questions must relate to the photosensory task of extracting time-of-day information from dawn and dusk [20,21]. During the dawn/dusk transition, light exposure changes in three key domains: the intensity; duration; and wavelength of light (Table 2). As these parameters change in a systematic manner, they could, in theory, be used by the circadian system to detect the precise phase of twilight [21]. However, each of these stimuli will be subject to considerable variation or “noise” (Table 2), and the consequences of this noise will depend upon the behavior of the organism and the environment in which it lives. There will be variation in the exposure to light, and individual responses to the light will depend upon the types of activity being undertaken, the light history of exposure, the age of the individual and of course the time of day. Reducing “noise” and the detection of a biologically relevant signal from background variation is a problem for all sensory systems, and much of the complexity of sensory systems reflects ways to achieve noise reduction. Color vision is an obvious example of noise reduction, providing a means of increasing the signal to noise ratio (contrast detection) of an object against its background, based upon the fact that different objects do not reflect the same wavelengths of light equally, and so can be detected using color vision.

From the discussion above we know that multiple photoreceptors and signaling pathways contribute to entrainment. However, we have limited knowledge regarding how different signals might be utilized. Some form of wavelength discrimination might be important, not just for contrast perception in vision, but also for the detection of twilight. At the dawn/dusk transition, there are very precise changes in the spectral environment (also see Section 2.3), primarily an enrichment of the shorter wavelengths (<500 nm) relative to the mid-long wavelengths (500–650 nm) [178,179]. If the circadian system were capable of detecting these changes by employing multiple photopigments to detect changes in the relative amounts of short and long wavelength light, then the phase of twilight could be determined very accurately. This idea was first proposed back in 2001 [21], and recently experiments have been undertaken that support this hypothesis [180]. The experimental approach simulated twilight conditions in the laboratory to explore whether mice can make use of these changes in wavelength for an estimation of the phase of twilight. Electrophysiological recordings the SCN showed that a sub-set of light-responsive neurons within the SCN are sensitive to changes in the spectral composition of daylight [90,180]. In addition to being sensitive to spectral changes, some neurons showed color-opponency in response to selective activation of short-wavelength sensitive photopigments versus long-wavelength sensitive photopigments. The color opponent process involves the processing of signals from cones and rods in an antagonistic manner, such that responses to one color of an opponent channel (e.g., blue) are antagonistic to those to the other color (e.g., red). That is, opposite opponent colors are never perceived together—there is no “blueish red”, only blue or red. Such a mechanism suggests that the SCN may indeed make use of this antagonistic effect to detect transitions from twilight to daylight [21,180].

The concept of separate NIF (melanopsin pRGCs) vs. IF (rods and cones) photoreceptors systems that engage in little cross-talk is now clearly wrong. A broad range of studies have shown that the light information reaching the SCN is derived from all retinal photoreceptor classes. As a result, SCN neurons can not only determine the amount of light but also the spectral quality of light for the precise detection of twilight [21].

### 2.10. Key Conclusions from Studies on Mice

- Light at twilight (dawn and dusk) is the key “zeitgeber” for the entrainment of circadian rhythms.
- The precise form of the phase response curve (PRC) varies but broadly light at dusk delays the clock (start activity later), whilst light at dawn advances the clock (start activity earlier).
- There is a suggestion that the PRCs of mice and humans differ with regard to the possession of a “dead zone.” However, methodological differences, especially the duration of the light pulses used, may account for these inconsistencies.
- The thresholds for entrainment vary between mouse strains (Table 1) and illustrate the point that there is variation in circadian photosensitivities within a single species.
- Mice lacking rods and cones show normal circadian entrainment. This finding demonstrated for the first time the existence of a “3rd ocular photoreceptor” within the mammalian eye.
- The 3rd ocular photoreceptor is based upon a network of photosensitive retinal ganglion cells (pRGCs).
- In addition to circadian entrainment multiple irradiance, detection tasks are mediated by the pRGCs.
- The photopigment of the pRGCs is melanopsin (OPN4) and has a peak spectral sensitivity in the “blue” part of the spectrum with a λ_max_ close to 480 nm.
- There are at least five different types of pRGCs based upon their anatomy and levels of melanopsin expression. The electrical properties of the pRGCs also vary and in some limited cases specific electrical responses can be linked to a specific pRGC sub-type.
- The single *Opn4* gene is alternately spliced, and the long and short isoforms are expressed at different levels in the pRGCs. This adds to the complexity of pRGC signaling.
- Phototransduction in pRGCs results in cellular depolarization and is very different from rod and cone phototransduction which leads to cellular hyperpolarization. Key details regarding pRGC phototransduction remain un-resolved.
- It remains unclear which sub-classes of the pRGCs project to different target regions of the brain and which pRGCs regulate specific behavioral and physiological responses.
- Rods (λ_max_~498 nm) and cones (M Cone λ_max_~508 nm; UVS~360 nm) do not project directly to the to pRGCs but modify their endogenous light response via the activation of inner retinal neurons.
- The sensory task of dawn/dusk (twilight) detection is complex in terms of: (1) the light signal itself (irradiance and wavelength); (2) individual exposure to the light signal; and (3) and individual responses to the light signal.
- It seems very likely that rods, cones and pRGCs interact to measure and integrate both the irradiance and wavelength of light at twilight to entrain the circadian system.
- The working hypothesis is that there is an integration of light signals within the pRGCs such that the rods are employed for dim light detection; cones are used for the detection of higher light intensities and for the integration of intermittent light exposure; and the pRGCs provide information regarding bright light over longer durations of exposure.

## 3. The Effects of Light on the Human Circadian System

**Figure 7:**
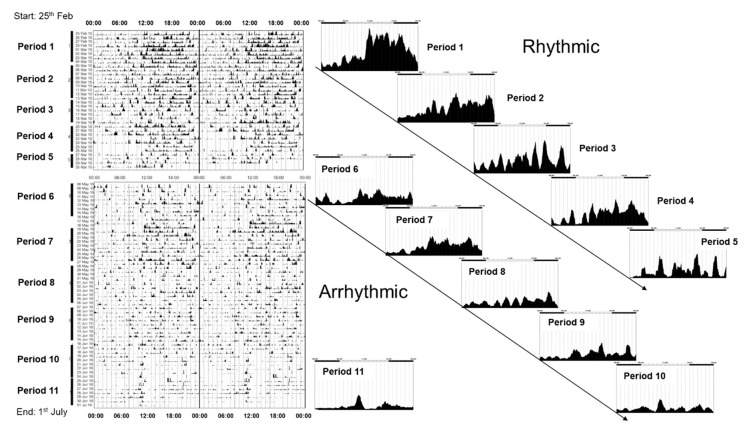
Data showing that the progressive loss of 24 h rhythmicity in a subject with a grade IV glioblastoma that infiltrated the anterior hypothalamus. Case study: Patient identifier (JJB), male, 67 years of age diagnosed with grade IV glioblastoma with progressive infiltration into the anterior hypothalamus. The Figure shows rest/activity recordings measured in the home environment using actigraphy and plotted on a 48 h time base from 25th February 2010 to the 1st July 2010, and the death of JJB. Actigraphy profiles are shown across eleven periods of analysis (times indicated on left of the actigraphy profile). Periodogram analysis of the activity profiles indicated diurnal rest/activity profiles close to a period of 24 h until 26th March 2010 (five periods); beyond this time the subject showed increasingly non-24 h (arrhythmic) behavior, as defined by periodogram analysis. Post-mortem analysis of the brain of JJB showed significant tumor (glioma) infiltration of the suprachiasmatic nuclei (SCN) and compression of this area of the brain due to basal brain swelling. The single peak of activity between ~10.00 and 12.00 seen in periods 10 and 11 corresponds to the daily visits to the home by a nurse. Unpublished data collected by Emma Cussans, Katharina Wulff, Olaf Ansorge and Russell Foster. Sincerest thanks are expressed to the family of JJB for their help and participation in the collection of these data during a very difficult time.

The organization of the human circadian system is broadly the same as the mouse, with a direct retinohypothalmic (RHT) projection from the eye to the SCN [181]. The importance of the integrity of the human SCN for 24 h patterns of behavior is illustrated in Figure 7.

Although a retinohypothalamic tract was identified definitively in humans in the early 1980s [181], the assumption was that human circadian rhythms are entrained primarily by social cues, with little if any role for light. There are several reasons for this. The first is that historically, the leading researcher investigating human circadian rhythms, and one of the forefathers of circadian rhythms research, Jürgen Aschoff, rejected the idea of photoentrainment in humans. Critically, Aschoff and colleagues had published high-profile papers suggesting that social cues can entrain human circadian rhythms; [182,183]. Subsequently, issues surfaced about the design of these experiments, largely relating to the use of self-selected lighting schedules and the use of bedside lamps. The view that humans are entrained by social cues was reinforced by animal studies. Work on rodents showed that the circadian system of mice is exquisitely sensitive to light such that a light/dark (L:D) cycle of only 0.01–0.1 lux (L) (e.g., Table 1) will entrain rest/activity cycles. Light at such levels was completely ineffective in humans. The first robust demonstration of photic entrainment used a L:D cycle of 5000 lux to achieve entrainment [184], and the discussion below focuses on the fact that humans do use light as their primary zeitgeber, but compared to rodents humans appear remarkably insensitive to light.

### 3.1. Identifying the 3rd Retinal Photoreceptor in Humans

All of the early experimental evidence from rodents (e.g., [26,27]) and humans (e.g., [185,186]) demonstrated that the circadian system is entrained by photoreceptors within the eye. However, a report in *Science* in 1998 suggested that bright light of 13,000 lux applied to the popliteal region (skin behind the knee) can shift circadian rhythms of body temperature and melatonin [187]. A media frenzy followed, and *Science* named the paper among the year’s top studies, and two patented treatments for sleep disorders soon followed. Nonetheless, some scientists challenged the findings at the time for pragmatic reasons; namely, that eye loss in humans blocks photoentrainment [188]. Other groups attempted to replicate these findings using various approaches [189,190,191] and then the methodologies were replicated precisely [192]. All failed to show that light applied to the popliteal region would phase shift the circadian system. Errors in the methodological approaches are now thought to have been the basis for the claim.

Following the original studies in “blind” mice [27], “blind” humans were examined. In contrast to animal studies, a major limitation for human work is the inability to correlate anatomically defined photoreceptor loss with light perception. In mice the retina can be examined histologically, whilst this is obviously not an option in humans. The first study to explore the impact of human eye disease upon NIF (non-image forming) responses to light, examined the ability of bright light to decrease plasma melatonin concentrations in eleven blind patients with no conscious perception of light, compared to six visually sighted subjects. Melatonin was suppressed following exposure to bright light in three sightless patients and in the normal subjects [193]. When two of these blind patients were examined further, by covering their eyes during light exposure, plasma melatonin did not decrease. Plasma melatonin was not suppressed following exposure to bright light in seven of the remaining blind patients; in the eighth, plasma melatonin was undetectable. These eight patients reported a history of insomnia [193]. A second study assessed sleep patterns in blind individuals and reported high levels of sleep disruption. Specifically, subjects with no conscious light perception showed more severe sleep disruption compared to those with some degree of light perception [194]. The conclusion from both studies was that the visual elements that mediate light-induced suppression of melatonin remain intact in some individuals lacking a conscious perception of light.

**Figure 8:**
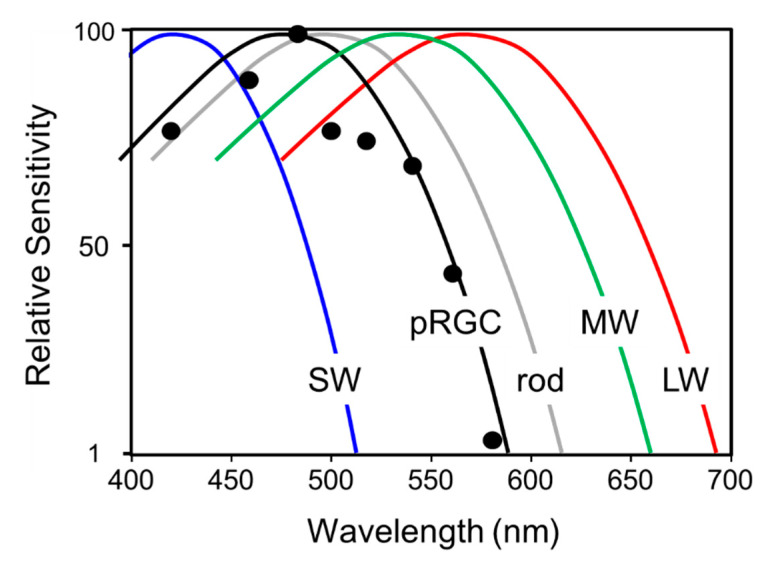
An action spectrum for pupillary constriction in a woman lacking functional rods and cones. Irradiance-response curves (IRCs) were generated at eight wavelengths for both eyes to define the action spectrum. The resulting action spectrum of pupil responses provided a poor fit to rod and cone photopigments (rod R2 = 0.35; short wave-sensitive (SWS) cone, mid wave-sensitive (MWS) cone, long wave-sensitive (LWS) cone R2 = 0). An optimum fit to the pupil response to light was provided by an opsin/vitamin A-based template with λ_max_ 476 nm (R2 = 0.89), corresponding closely to the pRGC system. The data shown were not corrected for pre-retinal lens absorption. When this correction was applied, the λ_max_ shifted from 476 nm to 480 nm [93].

These results [193,194] were consistent with studies in *rd/rd* mice [27], but had not demonstrated the existence of and additional class of ocular photoreceptor. This was eventually achieved, however, by examining the spectral sensitivity of NIF responses in two profoundly blind subjects who lacked functional rods and cones (one male, 56 year old; one female, 87 year old) [93]. Studies in the male subject showed that short-wavelength light would preferentially suppress melatonin, entrain circadian rhythms and enhance alertness compared to 555 nm light exposure, which is the peak sensitivity of human photopic vision. In a full action spectrum for pupillary constriction in the female subject, peak spectral sensitivity (λ_max_) was demonstrated to be at 480 nm, matching that of melanopsin-based pRGCs but not the λ_max_ of the rods and cones (Figure 8). The female subject was also able to recognize a short-wavelength stimulus (~480 nm) at threshold intensity, but not other wavelengths at the same intensity (equivalent photon flux). These data provided very strong evidence that melanopsin pRGCs regulate both circadian physiology and contribute to a rudimentary (subconscious) awareness of light [93].

Human melanopsin was first identified by Provencio and colleagues [63] and using in situ hybridization they showed that melanopsin expression was restricted to cells within the ganglion and amacrine cell layers of the primate retina. Like rodents, they found no expression in retinal photoreceptor cells. They also concluded that the anatomical distribution of melanopsin-positive retinal cells was similar to the pattern of cells known to project from the retina to the SCN [66]. A subset of primate retinal ganglion cells were shown to expresses melanopsin (Opn4), and the spectral sensitivity of human melanopsin followed. Determining the spectral sensitivity of mammalian melanopsins has been difficult. For example, attempts to measure the absorbance spectrum of primate melanopsins purified in vitro have provided inconclusive, with λ_max_ reported of 424 nm and 467 nm [72,195,196]. This problem was resolved by measuring physiological responses in HEK293 cells expressing human melanopsin. An action spectrum for light induced calcium responses predicted an opsin:vitamin A_1_ pigment that peaked at 479 nm [197], strikingly similar to the action spectrum for pupil constriction in an individual lacking functional rods and cones with a λ_max_ of 480 nm [93]. Collectively, the data suggested that human melanopsin-based pRGCs mediate non-rod, non-cone responses to light [196].

As discussed in Section 2.6 and Section 2.7, mice possess multiple pRGC subtypes (M1–M5), and a similar anatomical diversity is emerging in humans. Initial studies in primates, including humans, classified melanopsin immunoreactive RGCs as inner and outer stratifying cells, where outer stratifying cells represent M1 cells, and inner stratifying cells seem to represent M2 cells [60,198,199,200]. These early findings have now been updated by particularly elegant studies by Hannibal and colleagues who have identified M1, displaced M1, M2 and M4 cells [201]. They also found two other melanopsin pRGCs, named “gigantic M1 (GM1)” and “gigantic displaced M1 (GDM1).” They identified very few M3 cells and no M5 subtypes. Total cell counts from one human male and one female retina indicated that the human retina contains approximately 7283 ± 237 melanopsin pRGCs, which represents between 0.63% and 0.75% of the total number of RGCs. The melanopsin subtypes are not uniformly distributed, suggesting a level of functional specialization. Inputs to melanopsin RGCs were demonstrated from amacrine cells and directly from rod bipolar cells via ribbon synapses in the ON layer of the inner plexiform layer (IPL) and from dopaminergic amacrine cells and GABAergic processes in the outermost OFF layer of the IPL [201]. This study shows that humans, like mice, possess a heterogenic population of melanopsin pRGCs which are probably involved in mediating different behavioral and physiological responses to light. In addition, these pRGCs receive inputs from inner retinal neurons, strongly suggesting that rod and cone photoreceptors communicate, and likely modulate these photoreceptors [128].

### 3.2. The Intensity, Duration and Spectrum of Effective Light Stimuli

**Figure 9:**
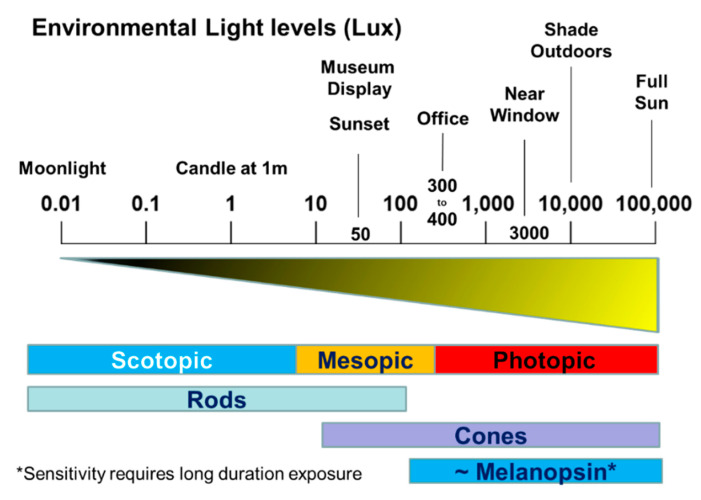
The human retina functions over a very wide range of light intensities. Scotopic vision is light detection by the rod photoreceptors of the eye under low light conditions. In the human eye cone photoreceptors are nonfunctional in low light and rods mediate scotopic vision. Photopic vision is light detection by the cone photoreceptors of the eye under bright light conditions. In humans and many other animals, photopic vision allows color perception, mediated by cone cells, and a significantly higher visual acuity and temporal resolution than available with scotopic vision. Mesopic vision is a combination of photopic vision and scotopic vision in low but not quite dark lighting situations and involves an input from both rod and cone photoreceptors. As light levels increase, and as rods become saturated, melanopsin photoreception is activated. Whilst this diagram gives some sense of the sensory thresholds for the different photoreceptor classes, it is also misleading in that it fails to take into consideration the differences in effective stimulus durations for the rods, cones and melanopsin-pRGCs. Rods and cones detect light over the millisecond range whilst melanopsin-based pRGCs require long duration exposure to light to elicit a biological response. See text for details.

Although much has been learned about the photic entrainment of circadian rhythms in rodents, studies in humans have lagged far behind. Beyond the fact that invasive physiological procedures are not possible in humans, the central problem has been that circadian studies require keeping individuals under controlled laboratory conditions for many days or even weeks. In addition, the accurate measurement of circadian rhythms in humans over extended time periods is very demanding on both the subject and researcher. Defining how the intensity, wavelength, duration and timing of light interact to regulate the human circadian system has been challenging. To provide some context to the discussion below, the approximate light levels within different environments and the visual sensitivities of the different photoreceptor classes have been illustrated in Figure 9.

### 3.3. The Impact of Different Light Stimuli on Circadian Entrainment 

#### 3.3.1. Field Studies and Natural Light Exposure

Field studies on humans exposed to natural light/dark cycles have demonstrated the importance of sunlight in human entrainment. An important study by Roenneberg and colleagues explored what zeitgebers entrain the human clock in real life by examining sleep/wake timing and chronotype across the same time zone. They make the point that dawn and dusk progress from east to west, which creates a continuum in sun rise and sun set across the surface of the planet. Thus, within the same Greenwich Mean Time (GMT)-defined time zone, dawn will be earlier in the east compared to the west, and the difference can be significant. For example, across the central European time zone which spans eastern Poland to western Spain, GMT defined midnight occurs almost one hour before mid-dark in Paris and more than 90 min earlier in Santiago de Compostela in Spain. Roenneberg and colleagues defined chronotype across the same time zone and addressed whether chronotype tracks social GMT-defined time or solar time. If social time acts as the primary zeitgeber, then there would be no change in chronotype in the population across the same time zone. However, they found that as you move from east to west across the same time zone, chronotype is earlier (relative to clock time) in the east compared to the west. Thus, the human circadian system seems to be predominantly entrained to sun time rather than social time [202]. In another paper, Roenneberg and colleagues also showed that the human circadian system tracks the seasonal change in photoperiod across the year, with mid-sleep occurring later in the winter compared to the summer [203].

The importance of natural light has also been demonstrated by Wright and colleagues. The first study examined the rest/activity timing of subjects over one week experiencing their normal daily routine with self-selected sleep schedules. The participants were exposed to an average of 979 ± 352 lux (±SD) during the waking hours of the week. This level of light is larger than reported for most individuals [204,205] and probably reflects a greater outdoor lifestyle and the sunny climate of the mountain-desert region of Colorado. Sleep/activity cycles were then compared to the effects of 1 week of outdoor camping during natural summer (14 h 40 min:9 h 20 min light/dark cycle) in tents and exposure to only natural light (i.e., sunlight and camp fires, no flashlights, no personal electronic devices, etc.) and self-selected sleep schedules. Average light levels during the week of natural light exposure (4487 ± 552 lux) were more than 4-fold greater compared to their normal daily routine. The result was an advance of sleep/wake and melatonin profiles by approximately two hours. The conclusion was that human sleep/wake timing (photoentrainment) under natural light-dark conditions tightly synchronizes to environmental time, and that in this regard, humans are just like other animals [206]. This study, using the same protocol, was then repeated during winter (9 h 20 min:14 h 40 min light/dark cycle). This time, subjects were exposed to light levels 13 times greater while winter camping (10,297 ± 2700 lux) compared to their normal daily routine (752 ± 424 lux). Sleep onset occurred approximately 2.5 h earlier during camping compared to home lighting, whereas wake time was similar in both environments. Thus, average sleep duration was approximately 2.3 h longer during winter camping (9.9 ± 0.4 h) compared to the modern electrical lighting environment (7.6 ± 0.5 h). The findings also provided evidence that the human circadian clock adapts to seasonal change under natural light-dark cycles and is timed later in the modern environment in both winter and summer [207]. Most recently, a study on university students from around the world showed that the late chronotypes (“owls”) were exposed to light in the evening (delaying light; see Figure 1) but experienced little light in the morning (advancing light). The net effect was to shift the body clock to a later time [208].

#### 3.3.2. Quantitative Measures of Circadian Responses under Artificial Light Stimuli

The early studies on the impact of light on the human circadian system used bright white artificial light (~5000 to 10,000 lux) of a long duration (approximately three to six hours) to simply demonstrate that light was capable of entraining human circadian rhythms; [184,209,210]. However, defining the threshold of entraining light stimuli by systematically varying the intensity and duration of the stimulus has only been undertaken rarely and never at the level of detail seen in rodents. The irradiance of light exposure will greatly influence the magnitude of the response. Such a relationship is called an “irradiance (intensity) response curve” or IRC [51]. Examples of IRCs from mice are shown in Figure 3 and Figure 4. Perhaps the most complete IRC in humans was undertaken by Zeitzer and colleagues [211]. In this study a single light pulse of 6.5 h and of varying irradiance (1 to 10,000 lux) was delivered in the early biological night (causing a phase delay) and the effect of this stimulus on phase shifting the rhythm in plasma melatonin and melatonin suppression was assessed. The findings showed that humans are highly responsive to the phase-delaying effects of light during the early biological night and that both the phase resetting response to light, and the acute suppressive effects of light on plasma melatonin follow a logistic dose-response curve. On the basis of the IRCs constructed, it was calculated that saturation of the phase-shifting response occurs at ~550 lux and saturation of the melatonin-suppression response is predicted to occur at ~200 lux. Zeitzer and colleagues [211] concluded that based upon previous published work [184] the human circadian system is much more sensitive to light than previously considered and that even small changes in light exposure during the late evening hours can significantly affect both plasma melatonin concentrations and the entrained phase of the human circadian pacemaker. The limitation of this conclusion is that the duration of the stimulus was not systematically varied in these studies. Whilst ~ 550 lux can saturate phase-shifting responses, this was achieved with a stimulus duration of 6.5 h. In this context, there has been only one detailed study that has explored stimulus duration in detail [212]. In these studies the circadian phase of the melatonin rhythm and melatonin suppression were assessed after a bright light pulse (~10,000 lux) of 0.2 h, 1.0 h, 2.5 h or 4.0 h duration. Per min of exposure, the 0.2 h duration was over five times more effective at phase delaying the circadian pacemaker (1.07 ± 0.36 h) compared with the 4.0 h duration (2.65 ± 0.24 h). Acute melatonin suppression and subjective sleepiness also had a dose-dependent response to light exposure duration [212]. These results provide strong evidence for a non-linear resetting response of the human circadian pacemaker to light duration, perhaps reflecting rod/cone photoreceptor inputs. It would be interesting to undertake similar experiments using lower irradiances of light, and light of different wavelengths, to construct an accurate understanding of the duration/intensity relationships of the human circadian system.

A direct comparison is problematic due to differing methodologies, but even taking this into account, a key and striking contrast between rodents and humans is the difference in the absolute sensitivity of photoentrainment. The question is: why? This may simply reflect the difference between being diurnal vs. nocturnal as a mammal. Diurnal mammals are exposed to light across the duration of the day whilst nocturnal mammals, emerging from their burrows at twilight, experience low levels of light for a relatively short period. As such, increased sensitivity to light at dawn and dusk would be an advantage. In humans there is also the additional problem of artificial light. Estimates vary but the controlled use of fire by *Homo erectus* is thought to have begun some 600,000 years ago. If our ancestors were as sensitive to light as rodents, firelight would have been a major disruptor of circadian rhythms. As a result, there would have been a major selective advantage to decrease the light sensitivity of the human circadian system. Recently, a mechanism has been discovered in mice which limits the phase shifting effects of light on the circadian system involving the kinase SIK1 [213]. Perhaps this pathway has been the evolutionary target for reduced photosensitivity in humans, and a comparison of the properties of SIK1 in mice and humans might be interesting.

Although most of the light stimuli used have been presented continuously, studies in mice suggested that intermittent light exposure can also result in phase shifts, and on the basis of known photoreceptor dynamics, cone photoreceptors seem to contribute to NIF responses to light in mice [176]. In a fairly recent study, this phenomenon was explored in humans, testing the hypothesis that exposure to alternating red light and darkness can enhance circadian resetting responses in humans by repeatedly activating cone photoreceptors. Circadian rhythms of melatonin, cortisol, body temperature and heart rate were assessed before and after exposure to six hours of continuous red light (631 nm, 13 log photons cm^−2^ s^−1^), intermittent red light (1 min on/off), or bright white light (2500 lux) (which served as a positive control), all delivered near the onset of nocturnal melatonin secretion (early evening). The study found that exposure to intermittent red light (1 min on/off) in humans did not result in stronger circadian phase resetting or melatonin suppression, as compared to exposure to continuous red light. Hence, contrary to the hypothesis of the paper, repeatedly activating cone photoreceptors did not enhance circadian or melatonin suppression responses to red light [214]. A potential criticism of the study is that the stimulus used, relatively dim intermittent red light (1 min on/off), is not a high contrast stimulus, and a stimulus that would not normally be very effective at selectively stimulating cones [215].

Other studies have shown that exposure to alternating cycles of bright white light (~10,000 lux) and dim light (<15 lux) elicited phase shifts similar in magnitude to continuous light [216], even when total illumination time was greatly reduced compared to continuous light exposure [217]. In this study a 6.5 h stimulus was presented during the early biological night (Figure 1) comprising continuous bright light, continuous very dim light or intermittent light consisting of six 15 min bright light pulses separated by 60 min of very dim light, and therefore only 23% of the continuous bright light stimulus. Significantly, the size of the phase delay was not significantly different between 6.5 h of continuous bright light vs. the intermittent light exposure. These and other findings [218], show that intermittent light is as, if not more, effective compared to continuous light in resetting human circadian rhythms, and so humans, like rodents [22,175,176], have the capacity to integrate light information separated by intervening periods of darkness.

#### 3.3.3. Quantitative Measures of Circadian Responses Using Colored Light Stimuli

Early studies explored the effects of colored light on the human circadian system to assesses whether the rods and/or cones (rods (λ_max_~498 nm); SWS cones (λ_max_~420 nm); MWS cones (λ_max_~534 nm); and LWS cones (λ_max_~564 nm)) might be implicated in entrainment. For example, human subjects were exposed to three consecutive days of five hours red light (220 photopic/22 scotopic lux) given in the late biological night (phase advancing pulse). The researchers estimated that this stimulus would be below the spectral sensitivity threshold of scotopic rod-based photoreception (an estimate that might be flawed as rod photoreceptors can respond to just a few photons [219]), yet of sufficient strength to activate a photopic cone-based photoreceptors. Exposure to this red-light light stimulus was able to just significantly phase shift the circadian rhythm of melatonin and implied a contribution of cone photoreceptors [220].

Following the detailed action spectrum studies on mice (Section 2.3), the field was stimulated to replicate these approaches as much as possible in humans and three studies on human subjects were undertaken in quick succession. Two similar studies defined an action spectrum for light-induced melatonin suppression in normally sighted subjects [221,222]. Both assessed the effects of long duration (30–90 min) light exposure on plasma melatonin suppression by constructing irradiance response curves across a range of wavelengths. The results suggested a photopigment with a λ_max_ between 459 and 469 nm. The third study measured the electroretinogram (ERG) of human subjects to define the action spectrum that drives sensory adaptation of the cone visual pathway over the 24 h day. The findings predicted the involvement of a single novel opsin photopigment with a maximum sensitivity at 483 nm [223]. In all three of these human action spectra it was concluded that the resultant data could only be described on the basis of a single novel opsin photopigment, quite distinct from the rods and cones: Thapan et al. [222] λ_max_ = 459 nm (range 457–462 nm); Brainard et al. [221] λ_max_ = 464 nm (range 446–477 nm); Hankins and Lucas [223] λ_max_ = 483 nm (range 479–487 nm). There is a notable variance of some 20 nm between the studies. Part of the difference probably reflects the ways in which the visual pigment templates were fitted [51], combined with differences in the approach used to correct for short wavelength adsorption by the lens. Indeed, if the three sets of data are compared on an equivalent log-normal scale, then all can be described by the same opsin photopigment [224]. Thus, it appears that a single opsin/vitamin photopigment regulates both melatonin suppression and the physiology of the primary visual pathway.

These action spectra were followed by a large number of spectral sensitivity studies (rather than full action spectra) that examined the effect of different wavelengths in phase-advancing [225] and phase-delaying [226,227,228,229] the human circadian system. Responses to a 6.5 h light stimulus at a λ_max_ of either 460 nm or 555 nm and of an equal photon flux were compared. Both phase-shifting and melatonin suppression were greater in response to 460 nm compared to 555 nm of light, and exposure to the 460 nm light increased alertness and reaction times, decreased lapses of attention and reduced sleepiness as measured by EEG [227]. More recently, Brainard and colleagues compared the effects of a 90 min exposure to 420 nm vs. 460 nm monochromatic light of varying irradiance on the suppression of melatonin. They found that 460 nm light was significantly more effective than 420 nm light [230]. These data would be consistent with the view that the short wavelength cones of humans (S Cones) with a λ_max_ of near 420 nm play a less important role in phase shifting than the longer wavelength photoreceptors (pRGCs, rods and long wavelength sensitive (LWS) cones) at the durations used.

The findings from these and other low-resolution experiments suggest that short wavelength (“blue”) light is significantly more effective at producing phase shifts than longer wavelengths, and that blue light is much more effective than broad spectrum white light on a quantal basis [225,227,228]. However, all of these studies lacked the resolution to define the contribution of the different photoreceptor pigments, especially between melanopsin (λ_max_ of ~480 nm) and rods (λ_max_ of ~500 nm), and cannot be used to demonstrate an exclusive role for melanopsin in human light responses. However, as discussed above (Figure 8), studies on an individual lacking functional rods and cones have been able to link non-image forming responses to light to melanopsin-based photoreception. In this individual an action spectrum for pupillary constriction exhibited a λ_max_ of 480 nm, matching that of melanopsin-based pRGCs but not that of the rods and cones. Furthermore, this subject was also able to correctly report a threshold short-wavelength stimulus (~480 nm) but not other wavelengths [93].

On the basis of the data available, and as discussed above, the working hypothesis is that in humans, like mice, there is an integration of light signals at the level of the pRGCs such that the rods are employed for dim light detection; the cones are used for the detection of higher light intensities and for the detection and integration of intermittent light exposure; and the pRGCs provide information regarding high levels of light over extended periods of exposure. A similar conclusion has been made regarding pRGC and rod/cone involvement in pupil constriction [231]. This concept of pRGC/rod/cone interactions also helps explain why a melanopsin-like response can be deduced from the action spectra published by Brainard and Thapan [221,222] that were obtained from individuals possessing all of their retinal photoreceptors. The action spectra in these studies were based upon light durations of 30 or 90 min If melanopsin is the channel that responds maximally to long duration light exposure, then the long duration light stimuli used in these action spectra would have enriched the response with melanopsin inputs.

#### 3.3.4. Exposure to Light-Emitting Electronic Devices

This section is prefaced with the observation that many researchers hold the mutually exclusive view that the human circadian system is both relatively insensitive to light, yet likely to be sensitive to the dim light emitted by electronic devices. The use of light-emitting devices immediately before bedtime has been a concern in some quarters because of the potential impact these devices may have on human circadian timing, not least because the light they emit is enriched in blue light [232,233]. Most of the studies have focused upon the impact of such devices on melatonin suppression. For example, Cajochen and colleagues [233] showed that a five hour exposure to a (white) light-emitting diode (LED) backlit computer screen significantly suppressed melatonin and enhanced performance compared to a non-LED backlit screen. Similar small effects were obtained by Figueiro [232] who showed that a two hour exposure to light from cathode ray tube computer screens induced a slight, but not statistically significant reduction in melatonin concentrations in college students. This study was then extended by Rea and colleagues using Apple iPads [234] who showed that melatonin levels were not significantly suppressed after a one hour exposure to the tablets, but this difference reached significance after two hours.

The most detailed study to-date compared reading a light-emitting (LE)-eBook in dim room light for approximately 4 h (between 18:00–22:00) over five consecutive evenings compared to reading a printed book under the same conditions. The light from the LE-eBook was approximately 31 lux (note–similar levels of light are used for constant routine CT protocols), and the light reflected from the printed book was approximately 1 lux. The LE-eBook emitted blue-enriched light, with a λ_max_ around 452 nm, compared to the printed book reflecting broad-spectrum white light. The paper states that reading a LE-eBook in the hours before bedtime decreased subjective sleepiness; decreased EEG delta/theta activity (sleep need); and whilst reading, suppressed the evening rise of melatonin. Further, LE-eBook use lengthened sleep latency; delayed the phase of the daily rhythm of melatonin; reduced sleep propensity; and impaired morning alertness. However, the specifics of the study are informative. The biggest effects were on melatonin, suppressing evening levels of melatonin by 55.12 ± 20.12%, whereas the print-book showed no suppression. Furthermore, dim light melatonin onset (DLMO) was approximately 1.5 h later after five days of LE-eBook use. In contrast, the effects on circadian phase and other aspects of sleep were barely detectable. Following LE-eBook use, participants took less than 10 min longer to fall asleep and had less than 10 min less rapid eye movement (REM) sleep. There was no difference between conditions in total sleep time, sleep efficiency or the duration of non-REM sleep [235]. As a result, some caution needs to be exercised when the authors state “that reading an LE-eBook in the hours before bedtime likely has unintended biological consequences that may adversely impact performance, health, and safety” [235].

A cross-platform computer program has been developed called “f.lux” that adjusts a computer screens color temperature according to location and time of day based upon local sunrise and sunset. The effect is to reduce the total irradiance and the blue light emission from a screen in the evening compared to the morning. The proponents of f.lux hypothesize that altering the color temperature of the display to reduce the prominence of blue light at night will improve the sleep and reduce circadian rhythm disruption. Although the developer provides a list of relevant research on the website—https://justgetflux.com/—the program itself has yet to be tested robustly to determine its efficacy [236]. In spite of this, f.lux has been widely and positively reviewed by technology journalists, bloggers and users.

### 3.4. The Impact of Light History

Studies in humans and other animals provide evidence that the responses of the circadian system to light are influenced by prior exposure to light and darkness (light history) [145,168,237,238,239,240,241]. Several laboratory studies have examined how prior light history might alter subsequent responses to a standard light stimulus [242,243]. Following a 6.5 h, 200 lux light exposure during the biological night, the degree of melatonin suppression was measured. For 15 h prior to the light stimulus, subjects were maintained in a background light that was very dim (approximately 0.5 lux) or brighter light at 200 lux, and at the same intensity as the light stimulus. Greater levels of melatonin suppression were achieved in subjects experiencing the 0.5 vs. 200 lux background light [242]. A subsequent study on phase-shifting responses to a light following a dim light vs. bright light background produced similar results [243]. Overall, the findings to date suggest that the light levels to which humans are exposed will impact upon the sensitivity to photic entrainment stimuli. Given that many studies have shown that humans are exposed to relatively low levels of bright light during the waking day indoors [244,245,246,247,248], these findings may have important practical implications for lighting design, and in the design of constant routine (CT) experimental paradigms.

### 3.5. The Impact of Age on Circadian Photosensitivity

The phase of circadian entrainment is determined by multiple factors, including genetic polymorphisms in key clock genes that influence chronotype [249]; overall health; and developmental changes [250], age and sex [251,252]. The most marked changes in chronotype occur in adolescence, where sleep timing becomes markedly delayed [253,254,255,256,257,258,259]. The delayed sleep/wake cycle of adolescents is partly driven by psychosocial factors, including peer pressure and media use; increased assertion of autonomy and reduced parental control [260,261]; and biological factors relating to developmental changes in the circadian system [262,263,264], and significantly, altered sensitivities to light [265]. In this study, melatonin suppression of low vs. moderate levels of light was assessed in mid-pubertal (9.1–14.7 years) and late to postpubertal (11.5–15.9 years) adolescents. The treatment involved 1 h light exposure at four light levels: 0.1 lux, 15, 150 and 500 lux. One group received evening light exposure beginning at 11:00 pm; a second group received morning light beginning at 3:00 am. The findings showed that the pre to mid-pubertal group showed significantly greater melatonin suppression to 15 lux (9.2 ± 20.5%), 150 lux (26.0 ± 17.7%) and 500 lux (36.9 ± 11.4%) during evening light exposure compared to the late to postpubertal group (−5.3 ± 17.7%, 12.5 ± 17.3% and 23.9 ± 21.7%, respectively; *p* < 0.05). No significant differences were seen between the groups in the early morning melatonin suppression. These data suggest that early pubertal children show greater sensitivity to evening light compared to postpubertal adolescents. Such an increased sensitivity to evening light in younger adolescents might be particularly disruptive to sleep regulation in this group [265].

Whilst there is evidence for increased photosensitivity of the circadian system during early adolescence, there is evidence for decreased photosensitivity in elderly humans [266]. Circadian phase was assessed under a constant routine. Subjects were exposed 6.5 h of broad-spectrum light stimulus (spanning ~2 lux to ~8000 lux) beginning in the early biological night, and circadian phase was reassessed. The results showed a significant dose–response relationship between irradiance and the phase shift of the melatonin rhythm, with evidence that sensitivity, but not the maximal response to light, differed from that of younger adults. These findings suggest an age-related reduction in the phase-delaying response to moderate light levels [266]. One possibility for this decline in photosensitivity might be related the development of ocular problems such as cataracts [267]. This was tested by examining sleep quality before and after cataract surgery and using either clear (UV blocking) or blue blocking lens replacements. After surgery at six months, sleep quality had improved in both groups, suggesting that increased lens transmission had improved sleep/wake timing. There was no statistical difference between the two lens types, suggesting that the reduction in short-wavelength light transmission had not affected entrainment [268]. The conclusion that blue blocking lens replacements will not have a significant impact upon the availability of light to stimulate the pRGCs is also independently supported by another study [269].

Based upon mouse studies, there are also likely to be major changes in the circadian system as we age. For example, studies have shown that the retinohypothalamic tract diminishes in aged mice and that light-induced gene expression (*c-fos*) in the SCN is reduced by ~50%. Whatever the mechanism, increasing light exposure in the elderly seems to have positive benefits on sleep/wake timing. For example, Van Someren and colleagues have shown that long-term daily treatment with bright vs. dim light in the elderly ameliorated symptoms of disturbed cognition, mood, behavior, functional abilities and sleep. In these studies, light exposure was manipulated by installing a large number of ceiling-mounted fixtures with Plexiglas diffusers containing an equal amount of Philips TLD 840 and 940 fluorescent tubes in the common living room. Lights were on daily between 09.00 and 18.00. The aim was an exposure of approximately 1000 lux, measured before the eyes in the gaze direction. In the control “dim” light group were exposed to light of around 300 lux. These researchers concluded that the long-term application of bright light (around 1000 lux) should be considered for use in care facilities for elderly individuals to improve health and well-being. Note that a study by Zee and colleagues indicated that the acute phase-shifting response to moderate or high-intensity broad spectrum light is not significantly affected by age [270].

### 3.6. Key Conclusions from Studies on Humans

- Light at twilight (dawn and dusk) is the key zeitgeber for the entrainment of human circadian rhythms and humans show different phases of entrainment under artificial (usually phase delayed) vs. natural light (usually phase advanced).
- The precise form of the human phase response curve (PRC) is debated but broadly light at dusk delays the clock (starting activity and sleep later the next day), whilst light at dawn advances the clock (start activity and sleep earlier the next day).
- Compared to mice, humans require light stimuli of a high irradiance (>100’s lux) and of a long duration (>30 min) to achieve entrainment, but the precise irradiance/duration relationships have yet to be defined.
- Humans lacking rods and cones show normal circadian entrainment. This finding demonstrated for the first time the existence of a “3rd ocular photoreceptor” within the human eye.
- On the basis of similarities with mice, the 3rd ocular photoreceptor appears to be based upon a network of photosensitive retinal ganglion cells (pRGCs).
- In addition to circadian entrainment, multiple irradiance detection tasks are mediated by the pRGCs in humans (e.g., alertness, pupil constriction, melatonin suppression).
- An action spectrum in a rodless/coneless individual suggests that the photopigment of the pRGCs is based upon melanopsin with a λ_max_ close to 480 nm. Such results contradict earlier studies suggesting that the λ_max_ was close to 460 nm.
- Emerging anatomical results show that there are multiple types of pRGCs in the human retina. There is no knowledge regarding the function or projections of the different pRGCs.
- There is anatomical evidence that rods (λ_max_~498 nm); SWS cones (λ_max_~420 nm); MWS cones (λ_max_~534 nm); and LWS cones (λ_max_~564 nm) communicate with the pRGCs via intermediate neurons of the retina. Studies in the primate retina show that these photoreceptors modify the endogenous light responses of the pRGC.
- The sensory thresholds and spectral sensitivities of the different photopigments overlap. As a result, the use of monochromatic light to selectively stimulate a specific photoreceptor channel is not possible. However, the more recent use of silent substitution approaches does provide a possible way forward [271]. Nevertheless, most studies suggest that long duration exposure to “blue” light is the most effective stimulus for entrainment.
- On the basis of behavioral studies it seems very likely that rods, cones and pRGCs interact to measure and integrate both the irradiance and wavelength of light at twilight to entrain the circadian system.
- The working hypothesis, with significant extrapolation from mouse studies, is that there is an integration of light signals within the pRGCs such that the rods are employed for dim light detection; cones are used for the detection of higher light intensities and for the integration of intermittent light exposure; and the pRGCs provide information regarding bright light over extended periods of exposure.
- An individual’s age, prior light history and genetics modify how light defines the phase of entrainment.

## 4. Future Experiments Relating to Entrainment of the Human Circadian System

Any future approaches to enhancing human circadian entrainment by using exposure to artificial light need to take into account the following considerations:

### 4.1. Measuring Effectiveness

Entrainment to the light/dark cycle occurs when the endogenous period of the circadian oscillator is phase shifted by the appropriate number of minutes/hours each day to equal that of the entraining light/dark cycle and is expressed by the simple equation:

τ − T + φΔ = 0 (entrainment)

where:

*Tau* (τ) = the intrinsic period of the clock;
T = the period of the entraining light/dark cycle;
φΔ = phase shift needed for τ = T.

The early reports suggested that the intrinsic period of the human circadian system was ~25 h [272]. This would therefore require a daily phase advance (+φΔ) of ~one hour to remain entrained to the solar day. Such shifts could easily be detected against the background “noise” of human assays of circadian entrainment (Table 2). More recently, however, the intrinsic period of the human circadian system has been revised to ~24.2 h with a range across the population from 23.81–24.31 h [273]. This is very important. If the average circadian period of humans is ~24.2 h, then the daily phase shift required for entrainment is about 0.2 h (about 12 min). Thus, on a day-to-day basis, relatively small phase shifts appear to be required to achieve entrainment. As a result, artificial lighting system-designed to enhance human circadian entrainment might only need to expose individuals to light of a relatively low irradiance (~100 lux range) and of a duration in the 60–120 min range. However, the big problem of using such stimuli would be the difficulty of demonstrating efficacy. An individual phase shift of ~12 min would be impossible to detect from the background “noise” using the current assays to measure human circadian rhythms.

### 4.2. Defining the Optimum Duration, Irradiance, Wavelength and Timing of Artificial Light Stimuli

Although the definitive experiments have not been undertaken, there is clearly a complex relationship between stimulus duration and intensity. The effectiveness of low levels of light can be greatly increased by increasing the duration of exposure to the stimulus. It also seem likely that the rods, cones and pRGCs interact for the regulation of entrainment, and the working hypothesis is that there is an integration of light signals such that the rods are employed for dim light detection; cones are used for the detection of higher light intensities and for the integration of intermittent light exposure; and the pRGCs provide information regarding bright light over extended periods of exposure. Furthermore, the sensory threshold and spectral sensitivities of the different photopigments overlap. As a result, using monochromatic light to selectively stimulate a specific photoreceptor channel is highly problematic. In addition, we do not fully understand how different photoreceptors interact, in either an additive or antagonistic manner or both depending upon context [274].

The timing of the stimulus is also absolutely critical. Light exposure around dusk will phase delay the circadian system (go to bed and get up later), whilst early morning light exposure will phase advance the circadian system (go to bed and get up earlier) (Figure 1). If artificial light is to be used to enhance human circadian entrainment, then we need to know in the real world when this light should be administered. For example, it seems that evening light exposure in the home environment contributes to a phase delay in the circadian system and a later chronotype [206,207]. As a result, artificial light delivered in the morning could be used to counteract this influence. Yet another level of complexity regarding the effectiveness of the intensity, duration, wavelength and timing of the light stimulus will be an individual’s age and prior light history.

### 4.3. Experimental Options and Future Approaches

For the reasons articulated above, trying to develop evidence-based artificial lighting to enhance human circadian entrainment for the stabilization of the sleep/wake cycle will be far from straightforward, particularly as essential information regarding how light interacts with the human circadian system is still lacking. For example, we have only a limited understanding of how light intensity and the duration of light exposure interact. Many experiments have simply used a “super saturating stimulus” of very bright light (>5000 lux) for many hours to measure an effect. To address this and allied issues would necessitate that individuals are maintained within the laboratory under a constant routine, and then exposed to light stimuli where duration and intensity of the light stimulus are varied systematically to show efficacy. This would both take time and require significant resources. In addition to gaining a better mechanistic understanding of how specific features of the light environment are perceived, we also need to understand how humans respond to dynamic light exposure in the “real world” were light intensity, duration, spectral quality and the time of exposure vary greatly. In parallel with CR experiments, it would seem highly desirable to undertake assessments of circadian entrainment in the real world in large numbers of individuals at different ages, involved in different occupations. Such an approach is now feasible because devices are becoming available that measure non-invasively, continuously and over an extended period of time (weeks) light exposure (irradiance, duration, wavelength, time of exposure) and individual circadian timing (sleep/wake and other measures of circadian phase). Such measurements, in relatively large numbers of individuals, would provide a means to identify how key features of natural light exposure interact to achieve entrainment (or not). With such baseline results, the development of explicit interventions could then be designed and tested in the field, addressing questions such as, “When should artificial light exposure be used—before work, at work or at home in the evening?” Because circadian monitoring would be over days, the effects of incremental advancing or delaying phase shifts would be apparent, addressing the issues raised in Section 4.1. Furthermore, such “natural experiments” would be relatively high throughput and low cost compared to the demands of CR within the laboratory. We propose that natural light experiments will provide a key way forward for both our understanding of how light is detected by the human circadian system and for defining what is required for the development of artificial lighting systems to enhance human circadian entrainment.

## Figures and Tables

**Table 1:** Three separate studies that compared the percentages of mice entrained to light/dark cycles of varying irradiance.

**(A) The Percentage of Animals Entrained to 12L:12:D in C57 Wildtype and C3H *rd/rd* Mice.**					
Strain	100 lux	10.0 lux	1.00 lux	0.10 lux	0.01 lux
C57 wildtype	100 (9)	100 (9)	87.5 (8)	85.7 (7)	83.3 (6)
C3H *rd/rd*	100 (12)	100 (18)	31.5 (19)	0 (17)	-
**(B) The Percentage of Animals Entrained to 12L:12:D in C57 Wildtype, C3H *rd/rd* and C3H +/+ Mice.**					
Strain	100 lux	10.0 lux	1.00 lux	0.10 lux	0.01 lux
C3H wildtype	100 (28)	100 (8)	50 (10)	12.5 (8)	0 (8)
C3H *rd/rd*	100 (27)	100 (7)	100 (8)	12.5 (8)	0 (10)
**(C) The Percentage of Animals Entrained to 16L:8D in C3H Wildtype and C3H *rd/rd* Mice.**					
Strain	100 lux	10.0 lux	1.00 lux	0.10 lux	0.01 lux
C57 wildtype	100 (12)	100 (14)	100 (14)	100 (10)	75 (8)
C3H *rd/rd*	100 (16)	100 (11)	93.8 (16)	23.5 (17)	5.6 (18)
C3H wildtype	100 (4)	100 (2)	31.5 (2)	0 (4)	0 (4)

In these experiments the impact of mouse strain on the threshold for entrainment was determined. (**A**) Results reprinted from Ebihara and Tsuji [34] showing the percentage of entrainment of C57 wildtype and C3H *rd/rd* mice to L:D 12:12 of varying irradiances (lux). (**B**) Extensions of the study by Ebihara and Tsuji [34] and by Argamaso-Hernan [39]. In this study the threshold for entrainment of C57 wildtype, C3H *rd/rd*, and C3H wildtype mice to L:D 12:12 was determined. Note that C57 wildtype mice can entrain to light of a lower irradiance than C3H wildtype mice, and that the thresholds for entrainment in C3H *rd/rd* and C3H wildtype mice are similar. (**C**) In this study the thresholds for entrainment of C3H wildtype and C3H *rd/rd* mice to L:D 16:8 was determined. Again, the thresholds for entrainment in C3H *rd/rd* and C3H wildtype mice are similar. In each experiment the number in brackets below the % denotes the numbers of animals used for the study [21].

**Table 2:** The major sources of variation associated with the light regulation of the circadian system, with reference to humans but applicable to most animals.

**Variation in the Stimulus** **(Intensity, Duration, Wavelength)**	
**Channel/Signal Noise**	Fluctuations in the light signal. e.g., Cloud cover; daylength/season.
**Environmental Noise**	Extraneous light signals. e.g., Starlight; moonlight; artificial lightning.
**Receptor Noise**	Molecular noise of the receptor pathway. e.g., Variation in external temperature; individual gene polymorphisms in the receptor pathway
**Variation in exposure & response to the stimulus** **(Type of Activity, Light History, Age, Time of Day)**	
**Sensory Adaptation**	Changing receptor thresholds. e.g., Receptor habituation; changes in pupil size; ocular pigment migration; circadian gated responses.
**Behavioral Noise**	Behavioral state. e.g., type and time spent in work vs home vs recreational environment.
**Developmental Noise**	Stage of development. e.g., Altered behavioral, physiological, biochemical responses with age; impact of disease.

At dawn and dusk, the quality of light varies in in terms of its intensity, duration and wavelength. As these parameters all change in a systematic way over twilight, such features could be used by the circadian system to detect the phase of the dawn/dusk transition. In addition though, each is subject to considerable sensory “noise,” and the impact of this noise will depend upon the organism and the environment in which it inhabits and will arise from variation in the exposure to light and variable responses to the light as a result of the types of activity being undertaken, the history of light exposure, the age of the individual [177] and of course the time of day (Figure 1). Some examples of the types of noise that might be expected to complicate photoentrainment are illustrated in the right-hand column.
